# Mathematical model describing CoViD-19 in São Paulo, Brazil – evaluating isolation as control mechanism and forecasting epidemiological scenarios of release

**DOI:** 10.1017/S0950268820001600

**Published:** 2020-07-20

**Authors:** H. M. Yang, L. P. Lombardi Junior, F. F. M. Castro, A. C. Yang

**Affiliations:** 1 Department of Applied Mathematics, State University of Campinas, General Hospital of the Medicine School of University of São Paulo, Campinas, Brazil; 2 Division of Allergy and Immunology, General Hospital of the Medicine School of University of São Paulo, Campinas, Brazil

**Keywords:** Epidemiological scenarios, new coronavirus, numerical simulations, pulses of release, unique pulse of isolation/quarantine

## Abstract

In São Paulo, Brazil, the first case of coronavirus disease 2019 (CoViD-19) was confirmed on 26 February, the first death due to CoViD-19 was registered on 16 March, and on 24 March, São Paulo implemented the isolation of persons in non-essential activities. A mathematical model was formulated based on non-linear ordinary differential equations considering young (60 years old or less) and elder (60 years old or more) subpopulations, aiming to describe the introduction and dissemination of the new coronavirus in São Paulo. This deterministic model used the data collected from São Paulo to estimate the model parameters, obtaining *R*_0_ = 6.8 for the basic reproduction number. The model also allowed to estimate that 50% of the population of São Paulo was in isolation, which permitted to describe the current epidemiological status. The goal of isolation implemented in São Paulo to control the rapid increase of the new coronavirus epidemic was partially succeeded, concluding that if isolation of at least 80% of the population had been implemented, the collapse in the health care system could be avoided. Nevertheless, the isolated persons must be released one day. Based on this model, we studied the potential epidemiological scenarios of release by varying the proportions of the release of young and elder persons. We also evaluated three different strategies of release: All isolated persons are released simultaneously, two and three releases divided in equal proportions. The better scenarios occurred when young persons are released, but maintaining elder persons isolated for a while. When compared with the epidemic without isolation, all strategies of release did not attain the goal of reducing substantially the number of hospitalisations due to severe CoViD-19. Hence, we concluded that the best decision must be postponing the beginning of the release.

## Introduction

Mathematical models based on a well-documented natural history of the disease allow us to understand the progression of viral infections. This understanding, in turn, permits forecasting epidemiological scenarios when control mechanisms are introduced aiming at the reduction or elimination of infections. In the case of coronavirus disease 2019 (CoViD-19), which is caused by severe acute respiratory syndrome coronavirus 2 (SARS-CoV-2), a strain of the RNA-based SARS-CoV-1, the possibility of obtaining scenarios is of fundamental importance. The reason is that in serious cases due to SARS-CoV-2 (new coronavirus) infection, immune cells overreact and attack the lung cell causing acute respiratory disease syndrome and possibly death. In general, the fatality rate in elder patients (60 years or more) is much higher than young patients (60 years or less) and under 40 years seems to be around 0.2% [1].

The new coronavirus can be transmitted by droplets that escape the lungs through coughing or sneezing and infect humans (direct transmission), or they are deposited on surfaces and infect humans when in contact with this contaminated surface (indirect transmission). This virus enters into susceptible persons through the nose, mouth or eyes, and infects cells in the respiratory tract, being capable to release millions of new viruses.

Currently, there is no vaccine, neither an effective treatment, although many drugs are under clinical trial. Hence, isolation is the main, if not unique, way of controlling the dissemination of this virus in a population aiming at the change in the disease propagation (this change is commonly known as the flattening curve of the epidemic). Nevertheless, this isolation arises an important question: are there reliable strategies to release these isolated persons aiming to avoid the retaking of its original progression of infection?

Many mathematical and computational models are used to describe the current new coronavirus pandemic. In mathematical models, there is a fundamental threshold (see [2]) called the basic reproduction number denoted by *R*_0_, which is defined as the secondary cases produced by one case introduced in a completely susceptible population. When a control mechanism is introduced, this number is reduced and is called as the reduced reproduction number *R*_r_. Ferguson *et al*. [3] proposed a model to investigate the effects of isolation of susceptible persons. They analysed two scenarios, called by them as mitigation and suppression, and predicted the numbers of severe cases and deaths due to CoViD-19 without control mechanism, and compared them with those numbers when control was introduced.

In this paper, we formulate a mathematical model based on the natural history of CoViD-19 to understand the dynamic (or trajectories of dissemination) of new coronavirus transmission, and this model can be used to forecast changes in the epidemic under intervention. Many countries adopted isolation to control the rapid dissemination of SARS-CoV-2. To assess the control of the CoViD-19 epidemic by isolation, the model considers pulse isolation and a series of pulses of release (see [4] for a series of pulses vaccination). The model is applied to describe the isolation adopted in São Paulo, Brazil, to control the CoViD-19 epidemic. The description of the epidemic means that the model parameters are fitted against data collected from São Paulo to provide the epidemiological scenario under isolation. However, these persons cannot be isolated indefinitely. Based on the description of the epidemiological scenario with isolation to control the SARS-CoV-2 transmission in São Paulo, we provide the epidemiological scenarios when the release will begin.

## Mathematical model

**Fig. 1.**
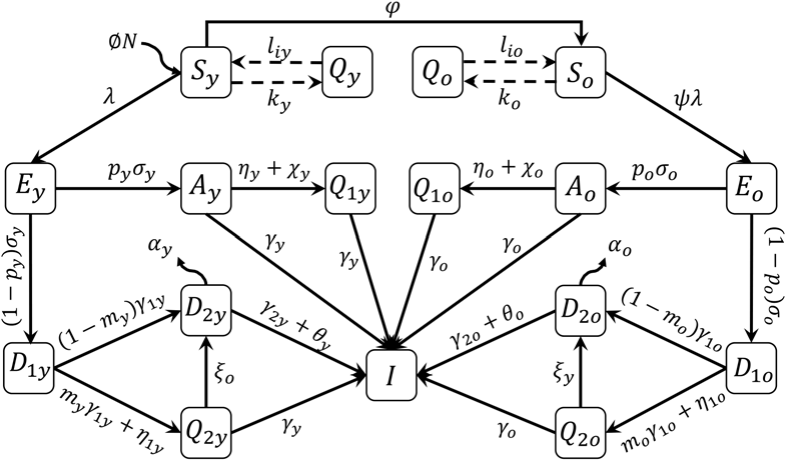
Flowchart of the new coronavirus transmission model with variables and parameters.

A mathematical model based on the natural history of CoViD-19 to describe epidemiological scenarios in São Paulo is detailed in the Supplementary Material. The flowchart shown in Figure 1 summarises the new coronavirus transmission model. The subscripts y and o stand for, respectively, young (60 years old or less) and elder (60 years old or more) subpopulations.

The model variables shown in Figure 1 are summarised in Table 1. Briefly, susceptible persons (*S*) are infected and enter into exposed and incubating the new coronavirus class (*E*). From this class, individuals enter into class *A* (asymptomatic) or class *D*_1_ (pre-diseased). From class *A*, individuals enter into class *Q*_1_ (isolated by a test) or class *I* (recovered). From class *D*_1_, individuals enter into class *Q*_2_ (isolated by a test) or class *D*_2_ (manifest severe CoViD-19). From classes *A*, *Q*_1_, *Q*_2_ and *D*_2_, individuals enter into recovered class *I*.

**Table 1.** Summary of the model variables for young (y)(*y*) and elder (o) subpopulations

Symbol	Meaning
*S*_*j*_	Susceptible persons
*Q*_*j*_	Susceptible persons in isolation
*E*_*j*_	Incubating new coronavirus persons
*A*_*j*_	Asymptomatic persons
*Q*_1*j*_	Asymptomatic persons caught by a test in isolation
*D*_1*j*_	Pre-symptomatic (pre-diseased) persons
*Q*_2*j*_	Pre-diseased persons caught by test in isolation
*D*_2*j*_	Severe CoViD-19 persons
*I*	Immune (recovered) persons

The model parameters shown in Figure 1 are described in Table 2 with respective values (for elder classes, values are between parentheses).

**Table 2.** Summary of the model parameters (*j* = y, o) and values (rates in per day, time in days and proportions are dimensionless). Some values are calculated (&), or varied (#), or assumed (*), or estimated (**)

Symbol	Meaning	Value
*μ*	Natural mortality rate	1/(75 × 365)[5]
*ϕ*	Birth rate	1/(75 × 365)*
φ	Ageing rate	6.7 × 10^−6^^&^
*σ*_y_(*σ*_o_)	Incubation rate	1/5(1/5)[1]
*γ*_y_(*γ*_o_)	Recovery rate of asymptomatic persons	1/10(1/11)[1]
*γ*_1y_(*γ*_1o_)	Infective rate of pre-diseased persons	1/4(1/4)[1]
*γ*_2y_(*γ*_2o_)	Recovery rate of severe CoViD-19	1/10(1/14)[1]
*ξ*_y_(*ξ*_o_)	Relapsing rate of pre-diseased persons	0(0)*
*α*_y_(*α*_o_)	Additional mortality rate	0.0018(0.009)**
*η*_y_(*η*_o_)	Testing rate among asymptomatic persons	0(0)*
*χ*_y_(*χ*_o_)	Voluntary isolation rate of asymptomatic persons	0(0)*
*η*_1y_(*η*_1o_)	Testing rate among pre-diseased persons	0(0)*
*k*_y_(*k*_o_)	Proportion of isolated susceptible persons	0.5(0.5)**
*l*_*i*y_(*l*_*i*o_)	Proportion released at time *t*_*i*_	0.33(0.33)^#^
*τ*^is^	Time of the introduction of the isolation	27
*τ*_1_	Time of the first release	72(10)
*θ*_y_(*θ*_o_)	Treatment rate	0(0)*
*β*_1y_(*β*_1o_)	Transmission rate due to asymptomatic persons	0.75(0.88)**
*β*_2y_(*β*_2o_)	Transmission rate due to pre-diseased persons	0.75(0.88)**
*ψ*	Scaling factor of transmission among elder persons	1.17**
*p*_y_(*p*_o_)	Proportion of asymptomatic persons	0.8(0.75)*
*m*_y_(*m*_o_)	Proportion of mild (non-hospitalised) CoViD-19	0.8(0.75)[6]

The introduction and establishment of the new coronavirus in São Paulo are described in the Supplementary Material. Based on the description of the epidemiological situation under isolation, we study the scenarios of release.

## Epidemiological scenarios of release

In the Supplementary Material, we presented the estimation of model parameters taking into account the data collected from São Paulo. The fitted values are *β*_y_ = 0.75, *β*_o_ = 0.88, *α*_y_ = 0.0018, *α*_o_ = 0.009 (all in per day) and *k* = 0.5, which are fixed hereafter unless explicitly cited. Taking into account the values given in Table 2, we solve numerically the system of equations provided in the Supplementary Material to obtain the epidemiological scenarios of the release. We stress the fact that we did not take into account the uncertainties in the parameter values given in Table 2, hence the scenarios of release must be taken as the forthcoming epidemic on the average.

Considering the isolation initiated on 24 March (*t* = 27) in São Paulo, we study the epidemiological scenarios of release beginning on 10 May. We assumed that all persons in isolation are considered susceptible (see Discussion). We simulate the beginning of release occurring at *t* = 72, but also at *t* = 56. We consider three strategies of release (between parentheses is the time of release beginning at *t* = 56): (strategy 1) all persons are released at *t* = 72 (56), (strategy 2) two releases equally distributed at *t* = 72 (56) and 79 (63) and (strategy 3) three releases equally distributed at *t* = 72 (56), 79 (63) and 86 (70). We also consider two regimes: (regime 1) equal proportions of release for young and elder persons and (regime 2) different proportions.

From the Supplementary Material, in the epidemic without isolation (*k* = 0), the peaks of the epidemic for young and elder persons are 5.02 × 10^5^ and 1.67 × 10^5^, which occur at *t* = 68. When 50% of persons are isolated (*k* = 0.5), the peaks of the epidemic for young and elder persons are 1.67 × 10^5^ (33%) and 5.84 × 10^4^ (35%), which occur at *t* = 88. The percentage between parentheses is the ratio of the peaks of the epidemic with and without isolation, peak (*k* = 0.5)/peak (*k* = 0).

In the scenarios of release, we present the curves of severe CoViD-19 cases *D*_2_ in the epidemic without isolation (*k* = 0), and the epidemic with isolation (*k* = 0.5) initiated at *t* = 27 but without release. These two epidemic curves are compared with the epidemic resulting from the release.

## Regime 1 – equal proportions of release for young and elder persons

Regime 1 considers the release of young and elder persons in the same proportion at the same time. In strategy 1, we consider a unique release with *l*_1*j*_ = 1, in strategy 2, two releases with *l*_1*j*_ = 0.5 and *l*_2*j*_ = 1, and in strategy 3, three releases with *l*_1*j*_ = 0.33, *l*_2*j*_ = 0.5 and *l*_3*j*_ = 1, for young (*j* = y) and elder (*j* = o) persons.

**Fig. 2.**
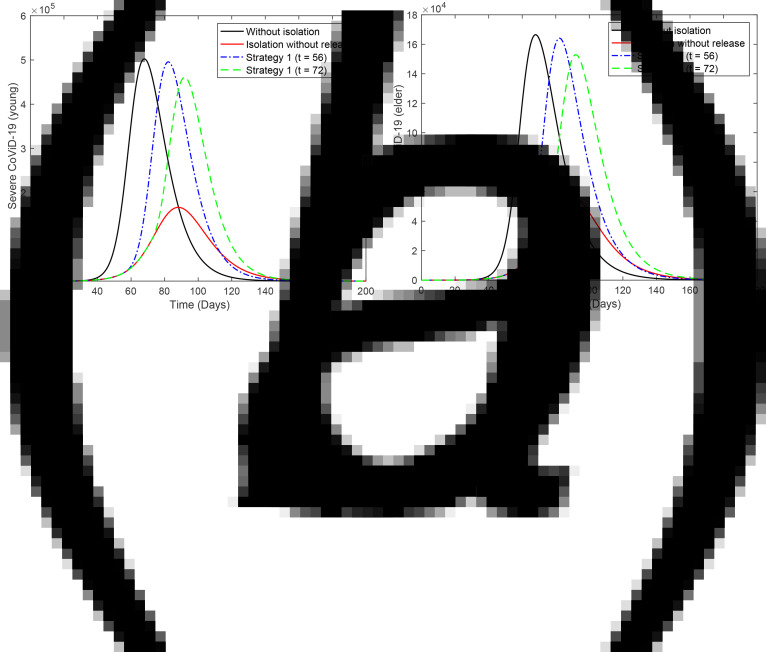
Curves of *D*_2_ without isolation (*k* = 0), with isolation (*k* = 0.5) initiated at *t* = 27 and release (strategy 1) beginning at *t* = 56 (dot and dashed) and 72 (dashed) for (a) young and (b) elder persons.

Figure 2 shows the curves of severe CoViD-19 cases *D*_2_ of the epidemic in young (a) and elder (b) subpopulations. For the release beginning at *t* = 56, according to strategy 1, the peaks of the epidemic for young and elder persons are, respectively, 4.96 × 10^5^ (99%) and 4.61 × 10^5^ (92%), which occur at *t* = 82. For the release beginning at *t* = 72, the peaks are 1.64 × 10^5^ (99%) and 1.53 × 10^5^ (92%), which occur at *t* = 92. Comparing the strategy of release beginning at *t* = 72 with the isolation without release, the peaks of the epidemic for young and elder persons are, respectively, anticipated in 6 and delayed in 4 days. The percentage between parentheses is the ratio of the peaks of the epidemic with and without isolation, peak (*k* = 0.5)/peak (*k* = 0).

**Fig. 3.**
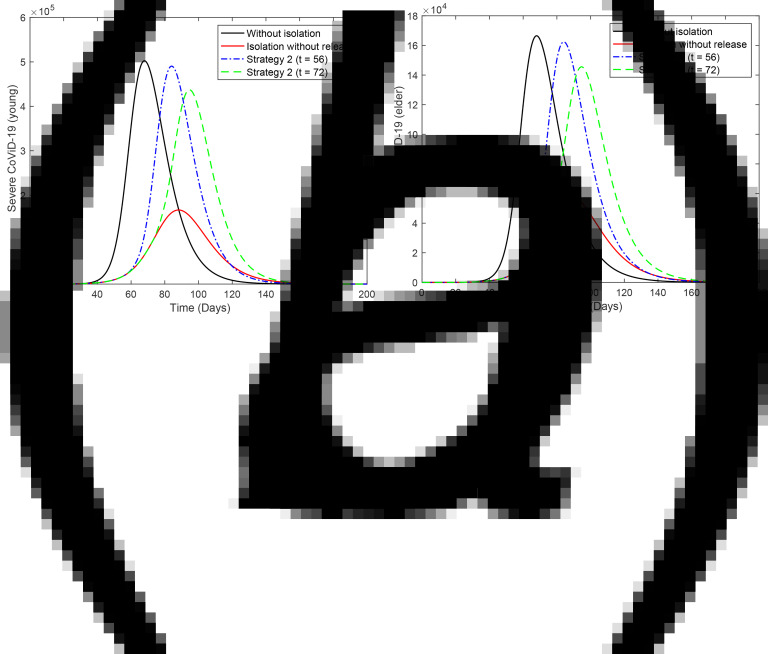
Curves of *D*_2_ without isolation (*k* = 0), with isolation (*k* = 0.5) initiated at *t* = 27 and release (strategy 2) beginning at *t* = 56 (dot and dashed) and 72 (dashed) for (a) young and (b) elder persons.

Figure 3 shows the curves of severe CoViD-19 cases *D*_2_ of the epidemic in young (a) and elder (b) subpopulations. For the release beginning at *t* = 56, according to strategy 2, the peaks of the epidemic for young and elder persons are, respectively, 4.90 × 10^5^ (98%) and 4.37 × 10^5^ (87%), which occur at *t* = 84. For the release beginning at *t* = 72, the peaks are 1.62 × 10^5^ (98%) and 1.46 × 10^5^ (87%), which occur at *t* = 94. Comparing the strategy of release beginning at *t* = 72 with the isolation without release, the peaks of the epidemic for young and elder persons are, respectively, anticipated in 4 and delayed in 6 days. The percentage between parentheses is the ratio of the peaks of the epidemic with and without isolation, peak (*k* = 0.5)/peak (*k* = 0).

**Fig. 4.**
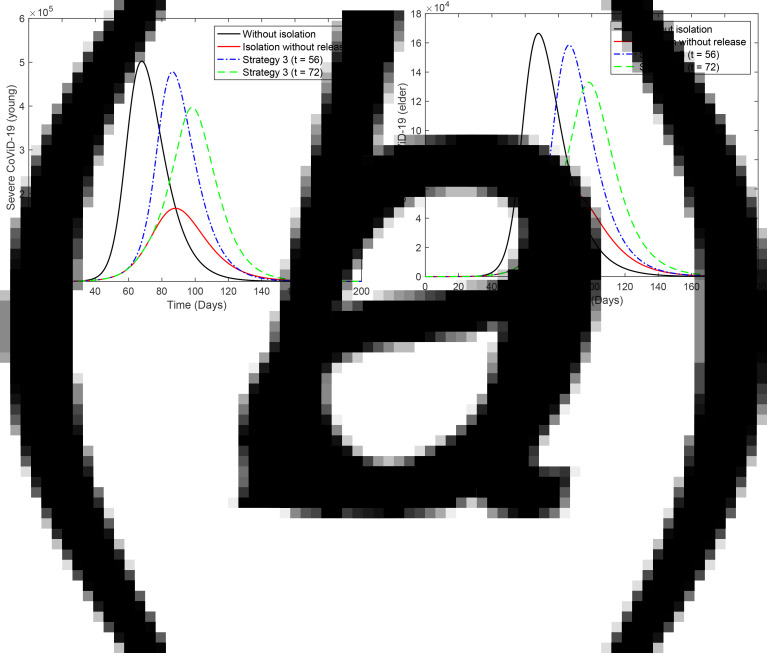
Curves of *D*_2_ without isolation (*k* = 0), with isolation (*k* = 0.5) initiated at *t* = 27 and release (strategy 3) beginning at *t* = 56 (dot and dashed) and 72 (dashed) for (a) young and (b) elder persons.

Figure 4 shows the curves of severe CoViD-19 cases *D*_2_ of the epidemic in young (a) and elder (b) subpopulations. For the release beginning at *t* = 56, according to strategy 3, the peaks of the epidemic for young and elder persons are, respectively, 4.78 × 10^5^ (95%) and 3.97 × 10^5^ (79%), which occur at *t* = 87. For the release beginning at *t* = 72, the peaks are 1.59 × 10^5^ (95%) and 1.33 × 10^5^ (80%), which occur at *t* = 98. Comparing the strategy of release beginning at *t* = 72 with the isolation without release, the peaks for young and elder persons are, respectively, anticipated in 1 and delayed in 10 days. The percentage between parentheses is the ratio of the peaks of the epidemic with and without isolation, peak (*k* = 0.5)/peak (*k* = 0).

From Figures 2 to 4, when the release of isolated persons is delayed in 16 days (*t* = 72), the peak of the epidemic is decreased and occurs later than the release beginning at *t* = 56. Observe that all releases in the three strategies are occurring in the increasing phase of the epidemic. (The last release occurs at *t* = 86, while the peak of the epidemic with isolation without release occurs at *t* = 88.) To avoid the higher peak of the epidemic after the release, the better strategy is postponing the beginning of the release.

Table 3 shows the values at *t* = 250 of accumulated CoViD-19 cases Ω, accumulated deaths Π, susceptible persons *S* and immune persons *I* for strategies 1, 2 and 3 with the first release occurring at *t* = 72. The percentages are calculated with respect to *k* = 0. In regime 1, the values of Ω, Π and *I* reach practically those attained by the epidemic without isolation (*k* = 0) at the end of the first wave of the epidemic. However, the number of susceptible elder persons is increased, but over 2900 cases at the end of the first wave of the epidemic without isolation. Hence, there is a very small difference among the three strategies, but delaying the release (beginning at *t* = 72 in comparison with 56) decreased the peak of the epidemic, suggesting that postponing the release to the decreasing phase of the epidemic may avoid an intense forthcoming epidemic.

**Table 3.** Regime 1: The values of Ω, Π, *S* and *I* at *t* = 250, for strategies 1, 2 and 3 for the release occurring at *t* = 72. The percentages are calculated with respect to *k* = 0

	Strategy 1			Strategy 2			Strategy 3		
	*y*	*o*	Σ	*y*	*o*	Σ	*y*	*o*	Σ
Ω (10^6^)	1.51	0.43	1.94	1.51	0.43	1.93	1.51	0.43	1.93
Π	26690	47610	74300	26680	47600	74280	26660	47570	74230
*S* (10^5^)	2.17	0.04	2.21	2.29	0.06	2.35	2.62	0.09	2.71
*I* (10^7^)	3.76	0.67	4.43	3.76	0.67	4.43	3.75	0.67	4.43
Ω (%)	99.93	99.88	99.92	99.87	99.86	99.87	99.80	99.81	99.80
Π (%)	99.96	99.87	99.91	99.93	99.85	99.88	99.85	99.79	99.81
*S* (%)	92.41	150.2	93.13	97.53	193.9	98.72	111.5	308.7	113.9
*I* (%)	100.1	99.90	100.0	100.0	99.88	100.0	99.92	99.82	99.91

### Regime 2 – different proportions of release for young and elder persons

Regime 2 deals with the release of young persons, but maintaining elder persons in isolation for a while. This regime presents quite similar results observed in regime 1 for the young subpopulation. Hence, we illustrate the release of young persons beginning at *t* = 72, but the release of the elder subpopulation will occur 21 days later, at *t* = 93. Observe that elder persons are released 5 days after the peak of the epidemic with isolation without release. The other illustration is the release of young persons beginning at *t* = 56, but elder persons are released at *t* = 77.

**Fig. 5.**
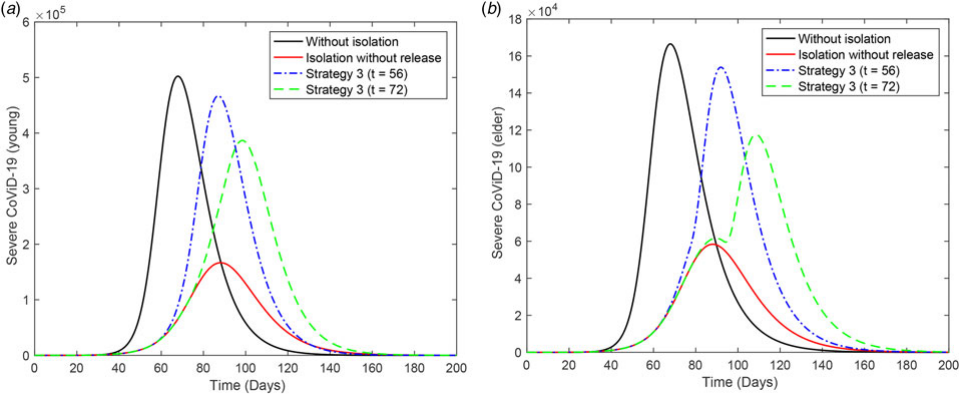
Curves of *D*_2_ without isolation (*k* = 0), with isolation (*k* = 0.5) initiated at *t* = 27 and release (strategy 3) of the young subpopulation beginning at *t* = 56 (dot and dashed) and 72 (dashed) for (a) young and (b) elder persons. All elder persons are released 21 days after the beginning of the release of young persons.

Figure 5 shows the curves of severe CoViD-19 cases *D*_2_ of the epidemic in young (a) and elder (b) subpopulations. The release of young persons occurs according to strategy 3, with the first release occurring at *t* = 56 (dot and dashed curve) or 72 (dashed curve). For the release beginning at *t* = 56, the peaks of the epidemic for young and elder persons are, respectively, 4.67 × 10^5^ (93%) and 3.87 × 10^5^ (77%), which occur at *t* = 87 and 98. For the release beginning at *t* = 72, the peaks for young and elder persons are 1.54 × 10^5^ (92%) and 1.18 × 10^5^ (71%), which occur at *t* = 92 and 109. The peaks of the epidemic for young and elder persons in this strategy of release, in comparison with those in isolation without release, are, respectively, anticipated in 1 and delayed in 10 days for release beginning at *t* = 56, while they are delayed in 4 and 21 days for release beginning at *t* = 72. Compared to Figure 4, the epidemic is slightly milder, but elder persons are infected later, which could be a desirable effect of isolation to protect the elder subpopulation under the higher risk of fatality induced by CoViD-19. The percentage between parentheses is the ratio of the peaks of the epidemic with and without isolation, peak (*k* = 0.5)/peak (*k* = 0).

In Figure 5(b), the release of 3.4 × 10^6^ susceptible elder persons at *t* = 93 resulted in the epidemic occurring after the peak of the epidemic with isolation without release. However, the peak of the epidemic decreased in comparison with regime 1 (see Figure 4(b)).

Now, we assess the epidemiological scenarios when elder persons are maintained isolated without release during the epidemic, but young persons are released according to the three strategies described in regime 1. This strategy is not easy to be implemented, but portrays the release of elder persons at the end of the first peak of the epidemic, for instance, at *t* = 200.

**Fig. 6.**
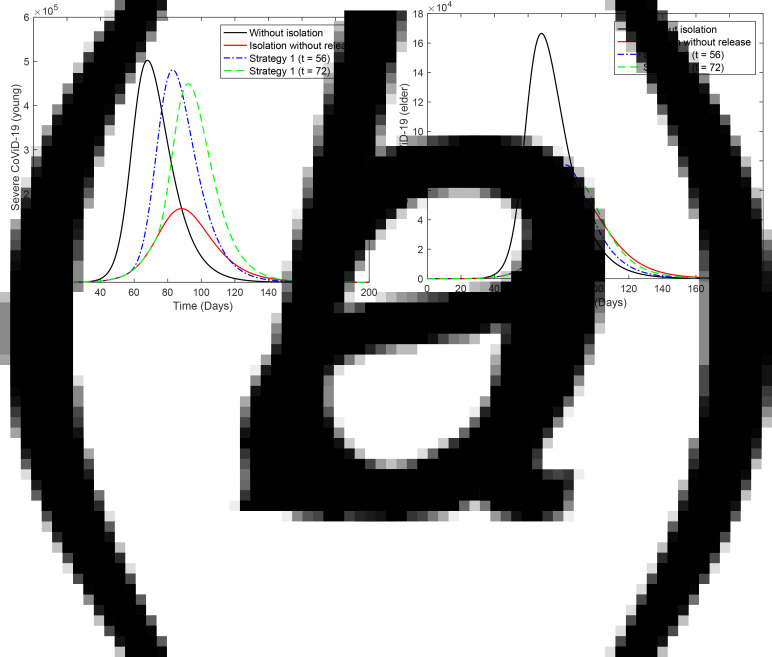
Curves of *D*_2_ without isolation (*k* = 0), with isolation (*k* = 0.5) initiated at *t* = 27 and release (strategy 1) of the young subpopulation beginning at *t* = 56 (dot and dashed) and 72 (dashed) for (a) young and (b) elder persons. Elder persons are not released.

Figure 6 shows the curves of severe CoViD-19 cases *D*_2_ of the epidemic in young (a) and elder (b) subpopulations. For the release beginning at *t* = 56, according to strategy 1, the peaks of the epidemic for young and elder persons are, respectively, 4.81 × 10^5^ and 4.49 × 10^5^, which occur at *t* = 83 and 92. For the release beginning at *t* = 72, the peaks for young and elder persons are 7.71 × 10^4^ and 6.62 × 10^4^, which occur at *t* = 83 and 89. The release of susceptible young persons enhanced the epidemic in course, and the consequence is a minor increase in the epidemic occurring in the circulating elder persons.

**Fig. 7.**
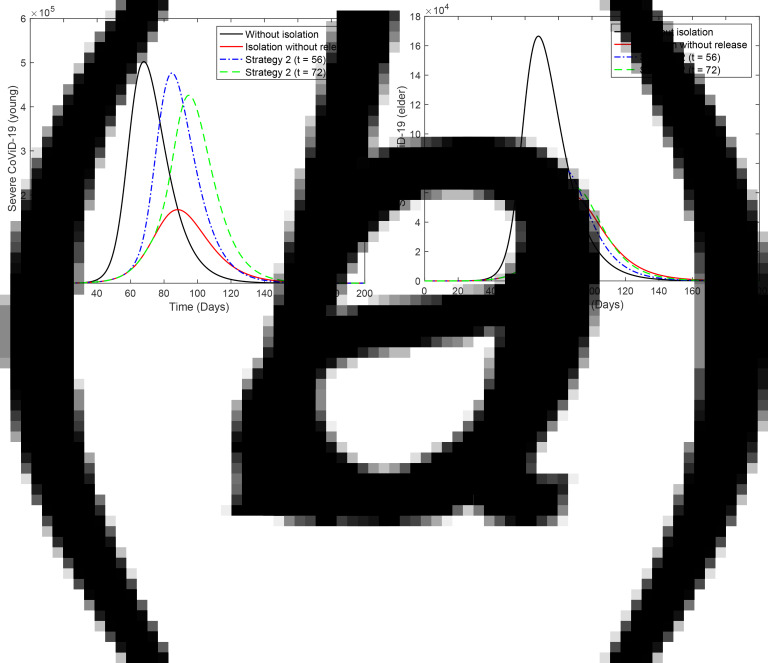
Curves of *D*_2_ without isolation (*k* = 0), with isolation (*k* = 0.5) initiated at *t* = 27 and release (strategy 2) of the young subpopulation beginning at *t* = 56 (dot and dashed) and 72 (dashed) for (a) young and (b) elder persons. Elder persons are not released.

Figure 7 shows the curves of severe CoViD-19 cases *D*_2_ of the epidemic in young (a) and elder (b) subpopulations. For the release beginning at *t* = 56, according to strategy 2, the peaks of the epidemic for young and elder persons are, respectively, 4.76 × 10^5^ and 4.26 × 10^5^, which occur at *t* = 85 and 95. For the release beginning at *t* = 72, the peaks for young and elder persons are 7.532 × 10^4^ and 6.35 × 10^4^, which occur at *t* = 84 and 90. The infection in the circulating elder persons was decreased in comparison with strategy 1.

**Fig. 8.**
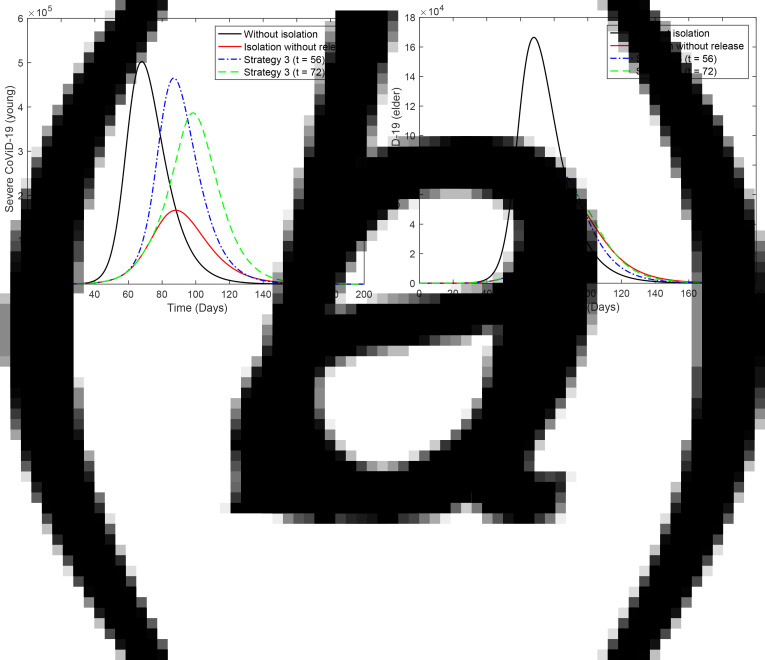
Curves of *D*_2_ without isolation (*k* = 0), with isolation (*k* = 0.5) initiated at *t* = 27 and release (strategy 3) of the young subpopulation beginning at *t* = 56 (dot and dashed) and 72 (dashed) for (a) young and (b) elder persons. Elder persons are not released.

Figure 8 shows the curves of severe CoViD-19 cases *D*_2_ of the epidemic in young (a) and elder (b) subpopulations. For the release beginning at *t* = 56, according to strategy 3, the peaks for young and elder persons are, respectively, 4.65 × 10^5^ and 3.87 × 10^5^, which occur at *t* = 87 and 98. For the release beginning at *t* = 72, the peaks for young and elder persons are 7.28 × 10^4^ and 6.158 × 10^4^, which occur at *t* = 86 and 89. For the release of young persons beginning at *t* = 72, we observe practically the absence of additional infection in circulating elder persons.

Table 4 shows the values at *t* = 250 of accumulated CoViD-19 cases Ω, accumulated deaths Π, susceptible persons *S* and immune persons *I* for strategies 1, 2 and 3 with the first release occurring at *t* = 72. The percentages are calculated with respect to *k* = 0. The values of Ω, Π, *S* and *I* for the young subpopulation reach practically those attained by the epidemic without isolation (*k* = 0) at the end of the first wave of the epidemic, as observed in regime 1. However, the epidemic in the circulating elder persons practically does not change compared to the epidemic without release. Again, there is a very small difference among the three strategies.

**Table 4.** Regime 2: The values of Ω, Π, *S* and *I* at *t* = 250, for strategies 1, 2 and 3 for the release occurring at *t* = 72. The percentages are calculated with respect to *k* = 0

	Strategy 1			Strategy 2			Strategy 3		
	y	o	Σ	y	o	Σ	y	o	Σ
Ω (10^6^)	1.51	0.21	1.72	1.51	0.21	1.72	1.51	0.21	1.72
Π	26660	23870	50530	26640	23870	50510	26600	23870	50470
*S* (10^5^)	2.67	0.03	2.70	2.90	0.03	2.93	3.45	0.029	3.48
*I* (10^7^)	3.75	0.34	4.09	3.75	0.34	4.09	3.74	0.34	4.08
Ω (%)	99.80	50.09	88.87	99.74	50.09	88.81	99.60	50.09	88.71
Π (%)	99.85	50.07	67.94	99.78	50.07	67.92	99.63	50.07	67.86
*S* (%)	113.8	95.07	113.6	123.5	96.02	123.2	147.1	98.20	146.5
*I* (%)	99.92	50.09	92.33	99.87	50.09	92.29	99.71	50.09	92.15

Comparing Tables 3 and 4, the release of young persons resulted in a very small change in the epidemic in the elder subpopulation. However, the isolated elder persons cannot be maintained indefinitely. We assess the release of all elder persons at four different times. The first case (release at *t* = 90) is just after the peak of the epidemic without release, the second case (release at *t* = 120) is in the descending phase of the epidemic, the third (release at *t* = 130) and the fourth (release at *t* = 140) cases are close to the end of the first wave of the epidemic. The isolated young persons are released according to strategy 3, with the release beginning at *t* = 56 or 72.

**Fig. 9.**
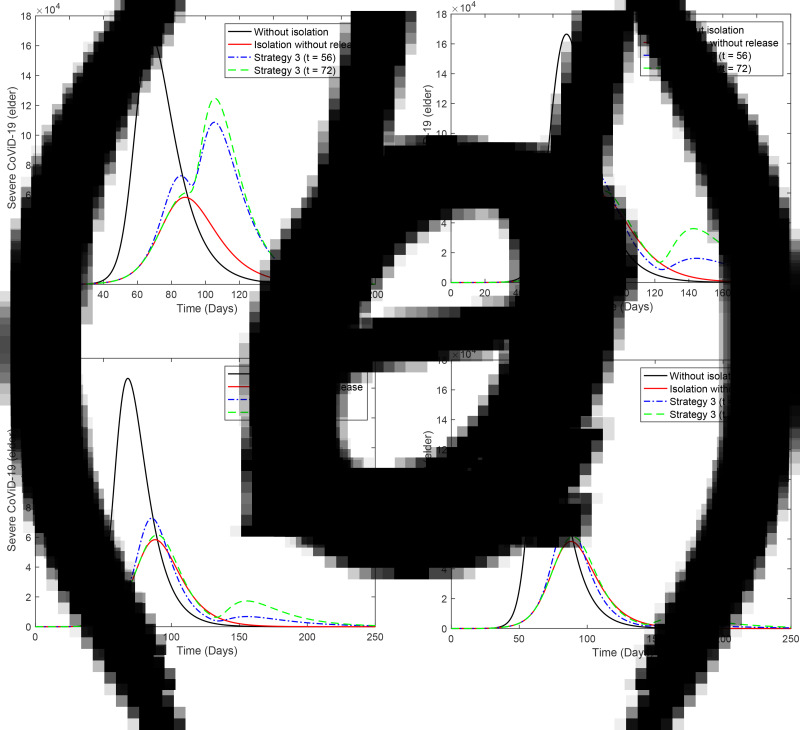
Curves of *D*_2_ for elder persons without isolation (*k* = 0), with isolation (*k* = 0.5) initiated at *t* = 27 and release (strategy 3) of young persons beginning at *t* = 56 (dot and dashed) and 72 (dashed). All elder persons are released at (a) *t* = 90, (b) 120, (c) 130 and (d) 140.

Figure 9 shows the curves of severe CoViD-19 cases *D*_2_ of the epidemic in the elder subpopulation, when elder persons are released at (a) *t* = 90, (b) 120, (c) 130 and (d) 140. The release of young persons is done according to strategy 3, with the release beginning at *t* = 56 (dot and dashed) or 72 (dashed).

As elder persons are released later, we observe the appearance of the second peak of the epidemic. The first peak of the epidemic is practically unchanged in comparison with the epidemic without release, but the second peak occurs later and its value is decreased. The release of young people increases the epidemic quickly in this subpopulation, but the elder subpopulation is relatively protected if the release occurs after *t* = 120. This is the desirable result of implementing the isolation, that is, the protection of those who are under increased CoViD-19 induced mortality, which is the case of elder persons infected with SARS-CoV-2. This strategy flattened the epidemic curve in the elder subpopulation, making more easier the efforts of hospitals to face the pandemic outbreak. However, Figure 9(d) shows a very small epidemic when elder persons are released, which indicates that a higher number of elder persons will be infected in the second wave of the epidemic.

**Fig. 10.**
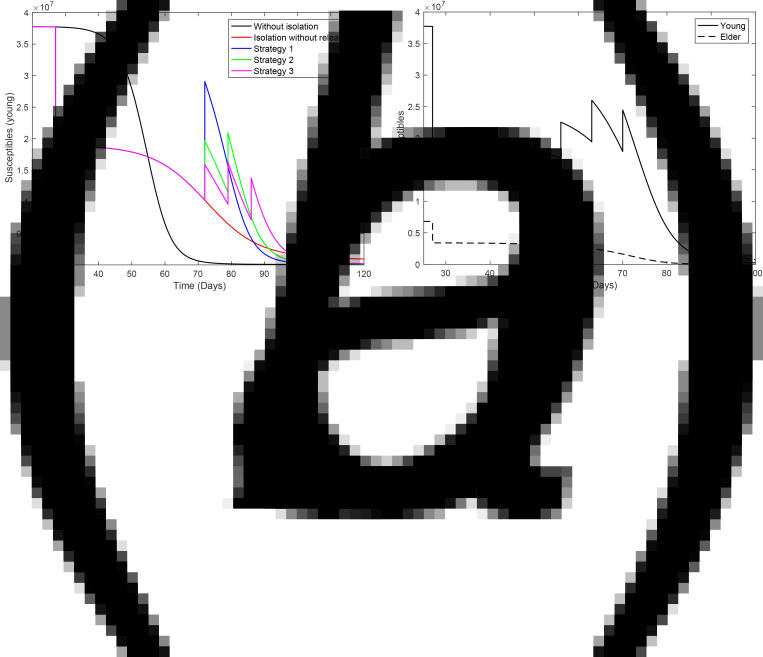
Pulse release of persons by regime 1 for (a) young persons and (b) regime 2. In (a) we show the strategy 3 including curves without (*k* = 0) and with (*k* = 0.5) isolation.

Figure 10 illustrates the pulses of release. Figure 10(a) shows the three strategies of regime 1 applied to the young subpopulation, and Figure 10(b) shows strategy 3 of regime 2 applied to the young and elder subpopulations. In Figure 10(a), the curves of the epidemic without (*k* = 0) and with (*k* = 0.5) isolation are included.

## Discussion

The system of equations described in the Supplementary Material was simulated to provide the epidemiological scenarios of isolation and further release in São Paulo, Brazil. The model parameters were estimated using the data collected from São Paulo (see Supplementary Material). To estimate the additional mortality rates, we used two methods, and we chose that providing more reliable values of deaths at the end of the first wave of the epidemic (see discussion in [7]). For instance, when we considered *R*_0_ = 4.1 and the higher estimated fatality rates were *α*_y_ = 0.08 and *α*_o_ = 0.4 (both in per day), we obtained 85% of deaths in the elder subpopulation at the end of the first wave of the epidemic.

In all scenarios of release, the peak of the epidemic was very high, which indicated the collapse of the health care system. However, if we increase the proportion of isolated persons above 80%, the health care system may not collapse. The number of severe CoViD-19 cases and the occupancy of beds in hospitals and ICUs can be related using the equations given in the Supplementary Material. To illustrate this relationship, we let *h*_1_ = *h*_1y_ = *h*_1o_ = 1 (at the beginning of the epidemic all cases are hospitalised), *h* = *h*_y_ = *h*_o_ = 0.3, 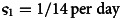 (2 weeks of hospital care) and 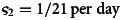 (3 weeks of ICU care) [6].

**Fig. 11.**
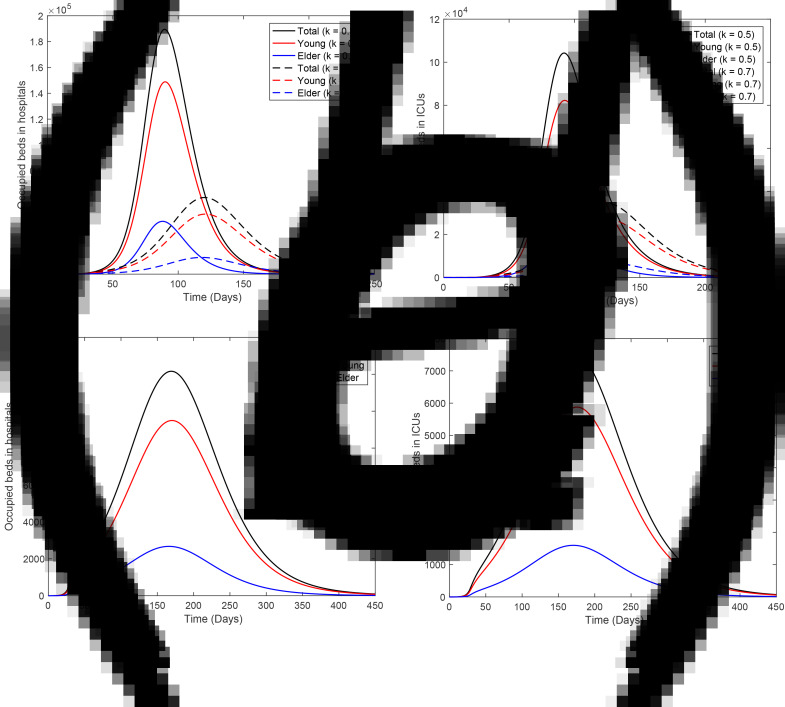
Number of occupied beds in (a) hospitals and (b) ICUs for *k* = 0.5 and *k* = 0.7 (dashed curves); and (c) occupied beds in hospitals and (d) ICUs for *k* = 0.8.

Figure 11 shows the number of occupied beds in hospitals (a) and ICUs (b) for *k* = 0.5 and *k* = 0.7 (dashed curves), and the occupied beds in hospitals (c) and ICUs (d) for *k* = 0.8. For *k* = 0.5, the peaks of occupied beds in hospitals and ICUs (this number is given within parentheses) for young and elder persons are, respectively, 1.49 × 10^5^ (8.22 × 10^4^) and 4.86 × 10^4^ (2.22 × 10^4^), occurring at *t* = 90 (92) and 88 (90). For *k* = 0.7, the peaks of the occupied beds in hospitals and ICUs (within parentheses) for young and elder persons are, respectively, 4.65 × 10^4^ (2.73 × 10^4^) and 1.29 × 10^4^ (0.74 × 10^4^), occurring at *t* = 121 (125) and 118 (121). For *k* = 0.8, the peaks of occupied beds in hospitals and ICUs (between parentheses) for young and elder persons are, respectively, 9500 (5880) and 2674 (1597), occurring at *t* = 170 (176) and 165 (169).

From Figure 11, for 80% of the isolated population, the peaks of occupied beds in hospitals and ICUs are, respectively, 12 174 and 7477. In this case, the health care system in São Paulo will not collapse if 19 651 patients could be admitted and treated in hospitals on 9 August (*t* = 165). However, this is not true for 70% of the population in isolation (treatment of 94 100 patients on 23 June, *t* = 118), as predicted by the public health authorities of São Paulo.

**Fig. 12.**
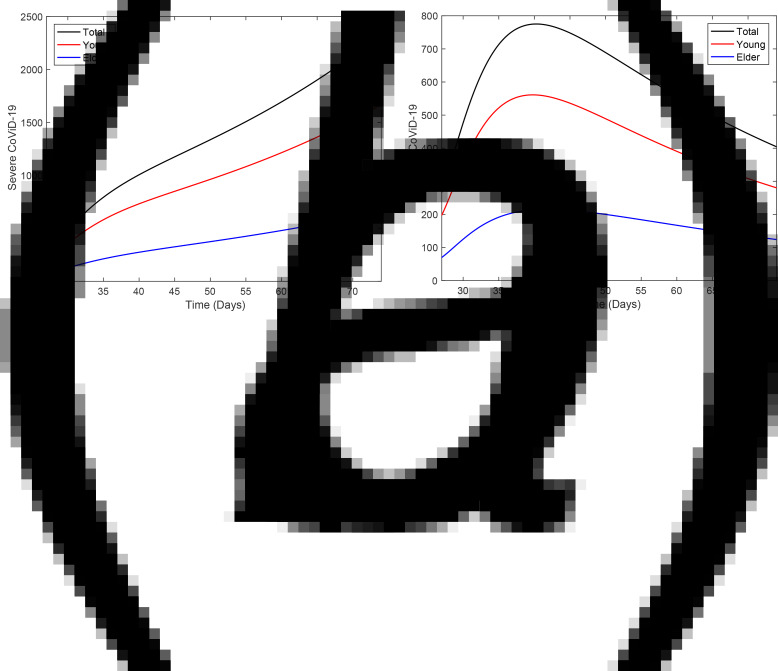
A new epidemic beginning in the isolated population, from *t* = 27 to 72. The curves of *D*_2y_, *D*_2o_ and *D*_2_ = *D*_2y_ + *D*_2o_ for (a) 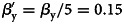 and (b)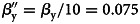.

When isolation is adopted during the epidemic, persons harbouring the new coronavirus can be found among isolated persons. In the epidemic with isolation in 50% of population, the number of persons in all classes at *t* = *τ*^is^ = 27 are
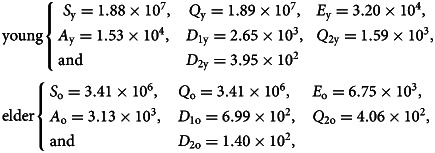
with *Q*_1y_ = *Q*_1o_ = 0 and *I* = 8.84 × 10^3^. To study the outbreak of the new coronavirus epidemic in the isolated population, we simulate the system of equations provided in the Supplementary Material taking the initial conditions at *t* = 27 given by: *S*_y_ = *Q*_y_ = 1.89 × 10^7^, *S*_o_ = *Q*_o_ = 3.41 × 10^6^, *Q*_1y_ = *Q*_1o_ = 0, and for all other variables, half of the corresponding values at *t* = 27. Figure 12 shows the curves of the epidemic (*D*_2y_, *D*_2o_ and *D*_2_ = *D*_2y_ + *D*_2o_) during the period of isolation for 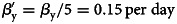 resulting in *R*_0_ = 1.4 (a), and 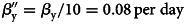 resulting in *R*_0_ = 0.7 (b). At *t* = 72, the number of severe CoViD-19 cases for young and elder persons are, respectively, 1597 and 635 for *R*_0_ = 1.4, and 294 and 129 for *R*_0_ = 0.7. When the epidemic triggered in the isolated population has the basic reproduction number below 1 (in our example, *R*_0_ = 0.7), the number of CoViD-19 cases at *t* = 72 is 423 (0.3%), and when *R*_0_ is doubled, this number is 2232 (1.8%), increased by 5.3 time. The percentage between parentheses is the ratio of the number of severe CoViD-19 cases in the isolated and circulating populations at *t* = 72. For this reason, we did not consider the transmission of SARS-CoV-2 in the isolated population.

**Fig. 13.**
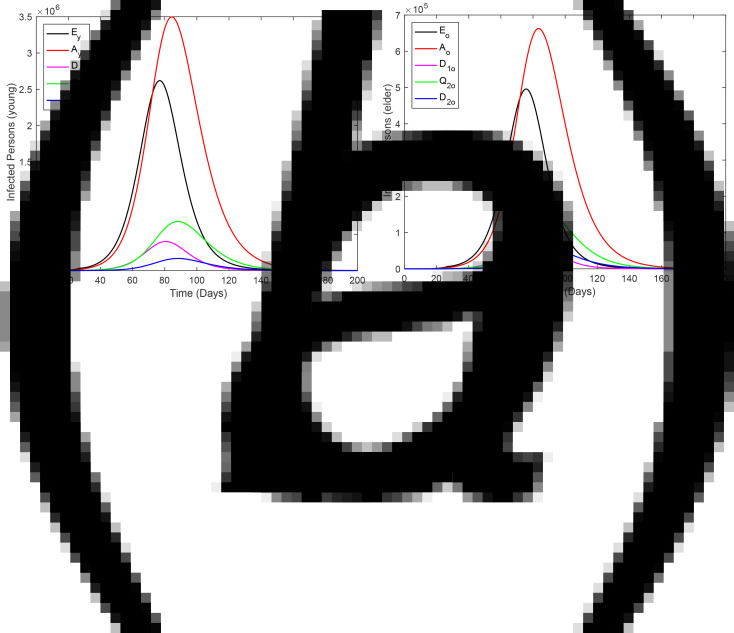
Curves of all persons harbouring the new coronavirus (*E*_*j*_, *A*_*j*_, *D*_1*j*_, *Q*_2*j*_ and *D*_2*j*_), *j* = y, o, for (a) young and (b) elder subpopulations.

Up to now, we showed only the curve of the severe CoViD-19 cases *D*_2_. Figure 13 shows all classes harbouring this virus (*E*_*j*_, *A*_*j*_, *D*_1*j*_, *Q*_2*j*_ and *D*_2*j*_, for *j* = y, o) for young (a) and elder (b) persons. The illustration corresponds to the epidemic with isolation without release. The classes *E*_*j*_ (incubating) and *A*_*j*_ (asymptomatic) have the majority of infected persons in the young and elder subpopulations.

**Fig. 14.**
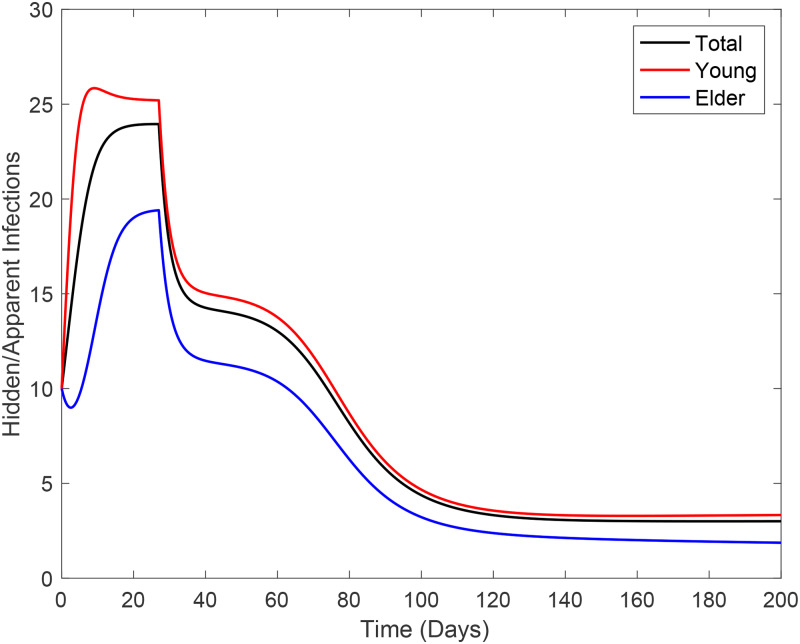
Curves of the ratio hidden:apparent for young, elder and total persons.

In Figure 14, we show the ratio hidden:apparent based on Figure 13. Those who incubate the new coronavirus and those who do not manifest symptoms are classified in the hidden category, and in the apparent category, we include those who manifest symptoms. Hence, the ratio is (*E* + *A* + *D*_1_):(*Q*_2_ + *D*_2_). At *t* = 0, the ratio is 10:1 for young and elder persons, given by the initial conditions.

Comparing Figures 13 and 14, as the epidemic evolves, the ratio increases quickly at the beginning, reaches a plateau during the increasing phase, and decreases during the declining phase, reaching another plateau at the ending phase of the first wave of the epidemic. Observe that the implementation of isolation at *t* = 27 avoided the quick decreasing in the ratio hidden:apparent. In the first plateau, the ratios are 18:1, 26:1 and 24:1 for, respectively, elder, young and total persons. The second plateau (5:1) is reached when the first wave of the epidemic is ending. The estimation of the ratio between hidden and apparent cases can help design mass testing to catch incubating and asymptomatic persons.

**Fig. 15.**
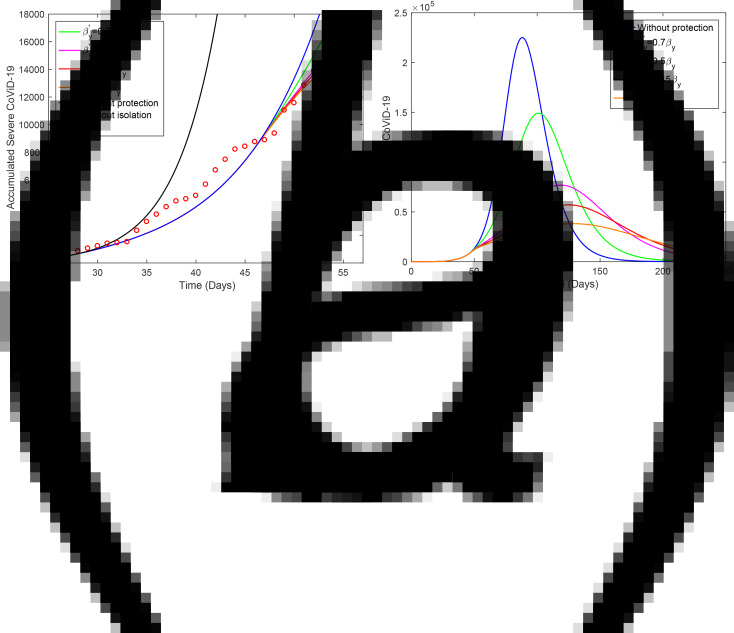
Curves of the accumulated CoViD-19 cases for 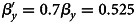, 0.5*β*_*y*_ = 0.375, 0.45*β*_*y*_ = 0.3375 and 0.4*β*_*y*_ = 0.3 (all in per day) and the (a) observed data. In (a) we show the curves of Ω with and without isolation. The curves of *D*_2_ extended from *t* = 0 until (b) 250.

In this paper, epidemiological scenarios were obtained aiming at the evaluation of isolation and subsequent release. However, from Figure S5(a) in the Supplementary Material, we observed a lowering in the curve of accumulated severe CoViD-19 compared with the observed data. Hence, we consider that at *t* = 45, 18 days after the beginning of isolation, protection actions were adopted by persons (face mask and social distancing) [8]. Figure 15(a) shows the curves of Ω for the reduced transmission rates 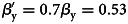, 0.5*β*_y_ = 0.38, 0.45*β*_y_ = 0.34 and 0.4*β*_y_ = 0.3 (all in per day), and the observed data. The better estimated is 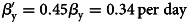. Figure 15(b) shows the curves of *D*_2_ extended from *t* = 0 until 250. We stress the fact that this evidence must be confronted with more data.

The question of individual (face mask and protection of eyes) and collective (social distancing) protective measures to avoid infection is left to future work.

## Conclusion

Many countries adopted the isolation of the population to control the rapid spreading of CoViD-19 to flatten the epidemic curve. We formulated a mathematical model based on the natural history of CoViD-19 encompassing young and elder subpopulations to study the transmission of SARS-CoV-2. As an example of application, we studied the effects of isolation adopted by São Paulo on the epidemic of CoViD-19.

Based on the model and using data collected from São Paulo, we estimated transmission rates, additional mortality (fatality) rates and proportion in isolation. We estimated *R*_0_ = 6.8 for the basic reproduction number, which is compatible with airborne infections (see [9] for the estimation of *R*_0_ = 6.7 for rubella infection). Currently, some authors found SARS-CoV-2 aerosol in some areas of the hospital environment, indicating the possibility of infection through air [10].

The model was simulated to provide the epidemiological scenarios when the release of the isolated population will begin. Using the estimated proportion *k* = 0.5 of isolated persons, we observed that in all three strategies of release for regime 1 (equal release of young and elder persons), the severe CoViD-19 cases approach those without isolation, but the peaks are delayed. However, for regime 2 (releasing young but maintaining elder persons), deaths due to CoViD-19 were reduced by half among elder persons and by 30% in all persons, which is a desirable result of the isolation, but hard to be implemented.

In all regimes and strategies of release, 50% of the population in isolation decreased the peak of the epidemic by around 10% and 25% for young and elder persons in comparison with the epidemic without isolation. These results showed that the release must be postponed. Indeed, on 8 May, São Paulo postponed the beginning of release to 1 June. Nevertheless, the simulations showed that if 80% or more persons were isolated, the desirable goal of reducing the severe CoViD-19 cases could be achieved.

In our model, severe CoViD-19 cases did not transmit the infection, considering that they were isolated in hospitals. However, during the treatment in hospitals, they are in close contact with health care workers. If severe cases of CoViD-19 release more amount of virus, a consequence could be increased transmission and more inhalation of the virus by health care workers, which is the reason to provide them with extremely secure equipment.

## Data Availability

The data that support the findings of this study are openly available in SP contra o novo coronavírus (Boletim completo) at https://www.seade.gov.br/coronavirus/, and SP contra o novo coronavírus (Adesão ao isolamento social em SP) at https://www.saopaulo.sp.gov.br/coronavirus/isolamento/.

## Supplementary material

For supplementary material accompanying this paper visit https://doi.org/10.1017/S0950268820001600. click here to view supplementary material
