# Supplementary material for: Mathematical model describing CoViD-19 in São Paulo, Brazil – evaluating isolation as control mechanism and forecasting epidemiological scenarios of release
Source: Epidemiol Infect. 2020 Jul 20;148:e155. doi: 10.1017/S0950268820001600 (PMC7378372; doi:10.1017/S0950268820001600)
Supplement: Supplementary file 1 [file S0950268820001600sup001.pdf]

Epidemiology and Infection

Mathematical model describing CoViD-19 in São Paulo State, Brazil – Evaluating isolation as control mechanism and forecasting epidemiological scenarios of release

H. M. Yang, L. P. Lombardi Junior, F. F. M. Castro, A. C. Yang

Supplementary Material

1 Introduction

The Supplementary Material is structured as follows. In Section 2, we introduce a model and the analysis of the corresponding model in terms of the fractions aiming at the calculation of the basic reproduction number R_0. In Section 2, we present the estimation of model parameters based on the data collected from São Paulo State.

2 Mathematical model

In this section, we present the mathematical model used in the main text, and the calculation of the basic reproduction number R_0 by analyzing the trivial equilibrium point in the steady state.

2.1 Formulation of a model

In a community where SARS-CoV-2 (new coronavirus) is circulating, the risk of infection is greater in elder than young persons, as well as elder persons are under the increased probability of being symptomatic and higher coronavirus disease 2019 (CoViD-19) induced mortality. Hence, the community is divided into two groups and composed of young (60 years old or less, denoted by subscript y) and elder (60 years old or more, denoted by subscript o) persons. The vital dynamic of this community is described by the per-capita rates of birth (ϕ) and death (μ).

For each subpopulation j ($j = y, o$), all persons are divided into eight classes: susceptible S_j, susceptible persons who are isolated Q_j, exposed E_j, asymptomatic A_j, asymptomatic persons who are caught by test and then isolated Q_{1j}, symptomatic persons at the initial phase of CoViD-19 (or pre-diseased, those who do not manifest disease) D_{1j}, pre-diseased persons caught by test and then isolated, plus mild CoViD-19 (or non-hospitalized) Q_{2j}, and symptomatic persons with severe CoViD-19 (hospitalized) D_{2j}. However, all young and elder persons in classes A_j, Q_{2j}, and D_{2j} enter into the same immune class I, the 17th class.

The natural history of CoViD-19 is the same for young ($j = y$) and elder ($j = o$) subpopulations. We assume that only persons in the asymptomatic (A_j) and pre-diseased (D_{1j}) classes are transmitting the virus, and other infected classes (Q_{1j}, Q_{2j} and D_{2j}) are under voluntary or forced isolation. Susceptible persons are infected according to $\lambda_j S_j$ (known as the mass action law [1]) and enter into class E_j, where λ_j is the per-capita incidence rate (or the force

of infection) defined by $\lambda_j = \lambda\left(\delta_{jy} + \psi\delta_{jo}\right)$, with λ being

$$\lambda = \frac{1}{N}\left(\beta_{1y}A_y + \beta_{2y}D_{1y} + \beta_{1o}A_o + \beta_{2o}D_{1o}\right), \tag{1}$$

where δ_{ik} is Kronecker delta, with $\delta_{ik} = 1$ if $i = k$, and 0, if $i \neq k$, and β_{1j} and β_{2j} are the transmission rates, that is, the rates at which viruses encounter susceptible people and infect them. After an average period $1/\sigma_j$ in class E_j, where σ_j is the incubation rate, exposed persons enter into the asymptomatic class A_j (with probability p_j) or pre-diseased class D_{1j} (with probability $1 - p_j$). After an average period $1/\gamma_j$ in class A_j, where γ_j is the recovery rate of asymptomatic persons, asymptomatic persons acquire immunity (recovered) and enter into immune class I. Another route of exit from class A_j is being caught by a test at a rate η_j and entering into class Q_{1j} and then, after a period $1/\gamma_j$, entering into class I. Possibly asymptomatic persons are in voluntary isolation, which is described by the voluntary isolation rate χ_j. The pre-diseased persons, after an average period $1/\gamma_{1j}$ in class D_{1j}, where γ_{1j} is the infective rate of pre-diseased persons, enter into non-hospitalized class Q_{2j} (with probability m_j) or hospitalized class D_{2j} (with probability $1 - m_j$). Hospitalized persons acquire immunity after a period $1/\gamma_{2j}$, where γ_{2j} is the recovery rate of severe CoViD-19, and enter into immune class I or die under the disease induced (additional) mortality rate α_j. Another route of exiting D_{2j} is by treatment, described by the treatment rate θ_j. After an average period $1/\gamma_j$ in class Q_{2j}, non-hospitalized persons acquire immunity and enter into immune class I, or enter into class D_{2j} at a relapsing rate ξ_j.

In the model, we consider a unique pulse in isolation at time $t = \tau_j^{is}$, described by $k_j S_j \delta\left(t - \tau_j^{is}\right)$, and m intermittent releases described by $\sum_{i=1}^{m} l_{ij} Q_j \delta\left(t - t_i\right)$, where $t_i = \tau_j^{is} + \sum_{w=1}^{i} \tau_{wj}$, $j = y, o$, and $\delta(x)$ is Dirac delta function, that is, $\delta(x) = \infty$, if $x = 0$, otherwise, $\delta(x) = 0$, with $\int_0^\infty \delta(x)\, dx = 1$. The parameters k_j and l_{ij}, $i = 1, 2, \cdots, m$, are the proportions in isolation and release of isolated persons, and τ_{wj} is the period between successive releases. All classes and parameters are summarized in Figure 1 shown in the main text.

Based on the above descriptions, the new coronavirus transmission model is described by the ordinary differential equations with $j = y, o$. The equations for susceptible persons are

$$\left\{\begin{array}{rcl} \dfrac{d}{dt}S_y & = & \phi N - (\varphi + \mu) S_y - \lambda S_y - k_y S \delta\left(t - \tau_y^{is}\right) + \sum\limits_{i=1}^{m} l_{iy} Q_y \delta\left(t - \tau_j^{is} - \sum\limits_{w=1}^{i} \tau_{wy}\right) \\ \dfrac{d}{dt}S_o & = & \varphi S_y - \mu S_o - \lambda\psi S_o - k_o S_o \delta\left(t - \tau_o^{is}\right) + \sum\limits_{i=1}^{m} l_{io} Q_o \delta\left(t - \tau_j^{is} - \sum\limits_{w=1}^{i} \tau_{wo}\right), \end{array}\right. \tag{2}$$

for infectious persons,

$$\left\{\begin{array}{rcl}\frac{d}{dt}Q_j & = & k_jS_j\delta\left(t-\tau_j^{is}\right)-\mu Q_j-\sum_{i=1}^{m}l_{ij}Q_j\delta\left(t-\tau_j^{is}-\sum_{w=1}^{i}\tau_{wj}\right)\\ \frac{d}{dt}E_j & = & \lambda\left(\delta_{jy}+\psi\delta_{jo}\right)S_j-\left(\sigma_j+\mu\right)E_j\\ \frac{d}{dt}A_j & = & p_j\sigma_jE_j-\left(\gamma_j+\eta_j+\chi_j+\mu\right)A_j\\ \frac{d}{dt}Q_{1j} & = & \left(\eta_j+\chi_j\right)A_j-\left(\gamma_j+\mu\right)Q_{1j}\\ \frac{d}{dt}D_{1j} & = & \left(1-p_j\right)\sigma_jE_j-\left(\gamma_{1j}+\eta_{1j}+\mu\right)D_{1j}\\ \frac{d}{dt}Q_{2j} & = & \left(m_j\gamma_{1j}+\eta_{1j}\right)D_{1j}-\left(\gamma_j+\xi_j+\mu\right)Q_{2j},\\ \frac{d}{dt}D_{2j} & = & \left(1-m_j\right)\gamma_{1j}D_{1j}+\xi_jQ_{2j}-\left(\gamma_{2j}+\theta_j+\mu+\alpha_j\right)D_{2j},\end{array}\right. \tag{3}$$

and for immune persons,

$$\begin{array}{rcl}\frac{d}{dt}I & = & \gamma_yA_y+\gamma_yQ_{1y}+\gamma_yQ_{2y}+\left(\gamma_{2y}+\theta_y\right)D_{2y}+\gamma_oA_o+\gamma_oQ_{1o}+\gamma_oQ_{2o}+\\ & & \left(\gamma_{2o}+\theta_o\right)D_{2o}-\mu I,\end{array} \tag{4}$$

where $N_j=S_j+Q_j+E_j+A_j+Q_{1j}+D_{1j}+Q_{2j}+D_{2j}$, and $N=N_y+N_o+I$ obeys

$$\frac{d}{dt}N=\left(\phi-\mu\right)N-\alpha_yD_{2y}-\alpha_oD_{2o}, \tag{5}$$

with the initial number of population at $t=0$ being $N(0)=N_0=N_{0y}+N_{0o}$, where the numbers of young and elder persons are N_{0y} and N_{0o}. If $\phi=\mu+\left(\alpha_yD_{2y}+\alpha_oD_{2o}\right)/N$, the total size of the population is constant.

The non-autonomous system of equations (2), (3), and (4) is simulated permitting intermittent interventions to the initial and boundary conditions. Hence, the equations for susceptible and isolated persons become

$$\left\{\begin{array}{rcl}\frac{d}{dt}S_y & = & \phi N-\left(\varphi+\mu\right)S_y-\lambda S_y\\ \frac{d}{dt}S_o & = & \varphi S_y-\mu S_o-\lambda\psi S_o\\ \frac{d}{dt}Q_y & = & -\mu Q_j,\end{array}\right. \tag{6}$$

for $j=y,o$, and other equations are the same.

For the system of equations (3), (4), and (6), the initial conditions (at $t=0$) are, for

$j = y, o$,

$$S_j\left(0\right) = N_{0j}, \quad X_j\left(0\right) = n_{X_j}, \quad \text{where} \quad X_j = Q_j, E_j, A_j, Q_{1j}, D_{1j}, Q_{2j}, D_{2j}, I, \tag{7}$$

and n_{X_j} is a non-negative number. For instance, $n_{E_y} = n_{E_o} = 0$ means the absence of exposed individuals (young and elder) at the beginning of the epidemic. We split the boundary conditions into isolation and release occurring at the same time for young and elder persons, that is, $\tau^{is} = \tau_y^{is} = \tau_o^{is}$ and $\tau_i = \tau_{iy} = \tau_{io}$, for $i = 1, 2, \cdots, m$, resulting in $t_i = \tau^{is} + \sum_{w=1}^{i} \tau_w$. A unique isolation at $t = \tau^{is}$ is described by the boundary conditions

$$S_j\left(\tau^{is+}\right) = S_j\left(\tau^{is-}\right)\left(1 - k_j\right) \quad \text{and} \quad Q_j\left(\tau^{is+}\right) = Q_j\left(\tau^{is-}\right) + S_j\left(\tau^{is-}\right) k_j, \tag{8}$$

plus

$$X_j\left(\tau^{is+}\right) = X_j\left(\tau^{is-}\right), \quad \text{where} \quad X_j = E_j, A_j, Q_{1j}, D_{1j}, Q_{2j}, D_{2j}, I, \tag{9}$$

with $\tau^{is-} = \lim_{t \to \tau^{is}} t$ (for $t < \tau^{is}$), and $\tau^{is+} = \lim_{\tau^{is} \leftarrow t} t$ (for $t > \tau^{is}$). The boundary conditions for a series of pulses released at t_i, for $i = 1, 2, \cdots, m$, are

$$S_j\left(t_i^+\right) = S_j\left(t_i^-\right) + l_{ij} Q_j\left(t_i^-\right) \quad \text{and} \quad Q_j\left(t_t^+\right) = \left(1 - l_{ij}\right) Q_j\left(t_i^-\right), \tag{10}$$

plus

$$X_j\left(t_i^+\right) = X_j\left(t_i^-\right), \quad \text{where} \quad X_j = E_j, A_j, Q_{1j}, D_{1j}, Q_{2j}, D_{2j}, I. \tag{11}$$

If $\tau_i = \tau$, then $t_i = \tau^{is} + i\tau$.

If isolation is applied to a completely susceptible population at $t = 0$, we have $S_j\left(0^+\right) = N_{0j}\left(1 - k_j\right)$ and $Q_j\left(0^+\right) = N_{0j} k_j$. However, if isolation is adopted at $t = \tau_j^{is}$ without a screening of persons harbouring the virus, then many of the asymptomatic persons could be isolated with susceptible persons, and the virus should be circulating through a restricted contact occurring in the household and/or neighborhood.

From the system of equations (3), (4), and (6), we derive the number of accumulated severe CoViD-19 cases, the number of accumulated deaths due to CoViD-19, and the number of occupied beds in hospitals.

The numbers of accumulated CoViD-19 (hospitalized) cases Ω_y and Ω_o are given by the exits from D_{1y}, Q_{2o}, D_{2o}, and Q_{2y} that are entered into classes D_{2y} and D_{2o}, that is,

$$\Omega = \Omega_y + \Omega_o, \quad \text{with} \quad \left\{ \begin{array}{l} \frac{d}{dt}\Omega_y = \left(1 - m_y\right)\gamma_{1y} D_{1y} + \xi_y Q_{2y} \\ \frac{d}{dt}\Omega_o = \left(1 - m_o\right)\gamma_{1o} D_{1o} + \xi_o Q_{2o} \end{array} \right. \tag{12}$$

with $\Omega_y(0) = \Omega_o(0) = 1$.

The number of accumulated deaths caused by severe CoViD-19 Π can be calculated from the hospitalized cases. This number of deaths is

$$\Pi = \Pi_y + \Pi_o, \qquad \text{with} \qquad \begin{cases} \frac{d}{dt}\Pi_y = \alpha_y D_{2y} \\ \frac{d}{dt}\Pi_o = \alpha_o D_{2o}, \end{cases} \tag{13}$$

with $\Pi_y(0) = \Pi_o(0) = 0$.

Finally, the number of occupied beds B during the epidemic is, for $j = y, o$,

$$B_1 = B_{1y} + B_{1o}, \quad \text{with} \quad \begin{cases} \frac{d}{dt}B_{1y} = h_{1y}\left(1 - h_y\right)\frac{d}{dt}\Omega_y - \left(\mu + \alpha_y + \varsigma_{1y}\right)B_{1y} \\ \frac{d}{dt}B_{1o} = h_{1o}\left(1 - h_o\right)\frac{d}{dt}\Omega_o - \left(\mu + \alpha_o + \varsigma_{1o}\right)B_{1o}, \end{cases} \tag{14}$$

for beds in hospitals with $B_{1y}(0) = B_{1o}(0) = 1$, and

$$B_2 = B_{2y} + B_{2o}, \quad \text{with} \quad \begin{cases} \frac{d}{dt}B_{2y} = h_{1y}h_y\frac{d}{dt}\Omega_y - \left(\mu + \alpha_y + \varsigma_{2y}\right)B_{2y} \\ \frac{d}{dt}B_{2o} = h_{1o}h_o\frac{d}{dt}\Omega_o - \left(\mu + \alpha_o + \varsigma_{2o}\right)B_{2o}, \end{cases} \tag{15}$$

for beds in ICUs, with $B_{2y}(0) = B_{2o}(0) = 0$. The fraction of severe CoViD-19 needing ICUs is h_j, and $1/\varsigma_{1j}$ and $1/\varsigma_{2j}$ are the average occupying time of beds in hospitals and ICUs for young and elder persons, where ς_{1j} and ς_{2j} are the discharging rates from hospitals and ICUs. The fraction h_{1j} is the severe CoViD-19 needing prolonged hospital care. The total number of occupied beds is $B = B_1 + B_2$.

The system of equations (2), (3), and (4) is non-autonomous. Nevertheless, the fractions of persons in each compartment approach the steady state (see next section 2.2, where all the equations cited below can be found). Hence, at $t = 0$, the basic reproduction number R_0 is obtained substituting s_y^0 and s_o^0 by N_{0y}/N_0 and N_{0o}/N_0 in equation (20), resulting in

$$R_0 = R_{0y}\frac{N_{0y}}{N_0} + R_{0o}\frac{N_{0o}}{N_0} = \left[p_y R_{0y}^1 + \left(1 - p_y\right)R_{0y}^2\right]\frac{N_{0y}}{N_0} + \left[p_o R_{0o}^1 + \left(1 - p_o\right)R_{0o}^2\right]\psi\frac{N_{0o}}{N_0},$$

where we used equations (26) and (27). We use the effective reproduction number R_{ef} given by equation (29), and study its variation during the epidemic. At $t = 0$, we have $R_{ef}(0) = R_0$, and as the time increases, R_{ef} decreases as susceptible persons decrease by infection. However,

at $t = \tau^{is}$ a pulse in isolation is introduced, and R_{ef} jumps down to $R_{ef}(\tau^{is+}) = R_r$ given by

$$R_r = R_0 \left[\frac{S_y\left(\tau^{is-}\right)(1-k_y)}{N_0} + \frac{S_o\left(\tau^{is-}\right)(1-k_o)}{N_0} \right], \tag{16}$$

where $S_y\left(\tau^{is-}\right)$ and $S_o\left(\tau^{is-}\right)$ are the numbers of susceptible young and elder persons at the time just before the introduction of isolation. At the i-th release time t_i, R_{ef} jumps up to $R_{ef}(t_i^+) = R_u(i)$ given by

$$R_u(i) = R_0 \left[\frac{S_y\left(t_i^-\right) + l_{iy} Q_y\left(t_i^-\right)}{N_0} + \frac{S_o\left(t_i^-\right) + l_{io} Q_o\left(t_i^-\right)}{N_0} \right],$$

and at the last time of release $t = t_m$, all isolated persons are released. When $t \to \infty$, we have $R_{ef} = 1$, and the new coronavirus epidemic reaches the equilibrium value $s^* = 1/R_0$, given by equation (28).

2.2 Analysis of the trivial equilibrium point

The system of non-linear differential equations (2), (3), and (4) is non-autonomous and non-constant population. To obtain an autonomous system of equations, we let $k_j = l_{ij} = 0$, $j = y, o$. To obtain equilibrium points, we use the fractions of individuals in each compartment, defined by

$$x_j = \frac{X_j}{N}, \quad \text{where} \quad X = S_j, Q_j, E_j, A_j, Q_{1_j}, D_{1_j}, Q_{2_j}, D_{2_j}, I,$$

for $j = y, o$, resulting in

$$\frac{d}{dt} x_j \equiv \frac{d}{dt}\frac{X_j}{N} = \frac{1}{N}\frac{d}{dt} X_j - x_j \frac{1}{N}\frac{d}{dt} N = \frac{1}{N}\frac{d}{dt} X_j - x_j (\phi - \mu) + x_j (\alpha_y d_{2y} + \alpha_o d_{2o}),$$

where we used the equation (5) for N. Hence, the system of equations (2), (3), and (4) become, for susceptible persons,

$$\left\{ \begin{array}{lcl} \frac{d}{dt} s_y & = & \phi - (\varphi + \phi) s_y - \lambda s_y + s_y (\alpha_y d_{2y} + \alpha_o d_{2o}) \\ \frac{d}{dt} s_o & = & \varphi s_y - \phi s_o - \lambda \psi s_o + s_o (\alpha_y d_{2y} + \alpha_o d_{2o}), \end{array} \right. \tag{17}$$

for infected persons,

$$\left\{\begin{array}{lcl}\frac{d}{dt}q_j & = & -\phi q_j + q_j\left(\alpha_y d_{2y}+\alpha_o d_{2o}\right)\\ \frac{d}{dt}e_j & = & \lambda\left(\delta_{jy}+\psi\delta_{jo}\right)s_j-\left(\sigma_j+\phi\right)e_j+e_j\left(\alpha_y d_{2y}+\alpha_o d_{2o}\right)\\ \frac{d}{dt}a_j & = & p_j\sigma_j e_j-\left(\gamma_j+\eta_j+\chi_j+\phi\right)a_j+a_j\left(\alpha_y d_{2y}+\alpha_o d_{2o}\right)\\ \frac{d}{dt}q_{1j} & = & \left(\eta_j+\chi_j\right)a_j-\left(\gamma_j+\phi\right)q_{1j}+q_{1j}\left(\alpha_y d_{2y}+\alpha_o d_{2o}\right)\\ \frac{d}{dt}d_{1j} & = & \left(1-p_j\right)\sigma_j e_j-\left(\gamma_{1j}+\eta_{1j}+\phi\right)d_{1j}+d_{1j}\left(\alpha_y d_{2y}+\alpha_o d_{2o}\right)\\ \frac{d}{dt}q_{2j} & = & \left(\eta_{1j}+m_j\gamma_{1j}\right)d_{1j}-\left(\gamma_j+\xi_j+\phi\right)q_{2j}+q_{2j}\left(\alpha_y d_{2y}+\alpha_o d_{2o}\right)\\ \frac{d}{dt}d_{2j} & = & \left(1-m_j\right)\gamma_{1j}d_{1j}+\xi_j q_{2j}-\left(\gamma_{2j}+\theta_j+\phi+\alpha_j\right)d_{2j}+d_{2j}\left(\alpha_y d_{2y}+\alpha_o d_{2o}\right),\end{array}\right. \quad (18)$$

and for immune persons,

$$\begin{array}{lcl}\frac{d}{dt}i & = & \gamma_y a_y+\gamma_y q_{1y}+\gamma_y q_{2y}+\left(\gamma_{2y}+\theta_y\right)d_{2y}+\gamma_o a_o+\gamma_o q_{1o}+\gamma_o q_{2o}+\left(\gamma_{2o}+\theta_o\right)d_{2o}-\phi i+\\ & & i\left(\alpha_y d_{2y}+\alpha_o d_{2o}\right),\end{array} \quad (19)$$

where λ is the force of infection given by equation (1) re-written as

$$\lambda=\beta_{1y}a_y+\beta_{2y}d_{1y}+\beta_{1o}a_o+\beta_{2o}d_{1o}.$$

The system of equations (17), (18), and (19) obeys

$$\sum_{j=y,o}\left(s_j+q_j+e_j+a_j+q_{1j}+d_{1j}+q_{2j}+d_{2j}\right)+i=1,$$

which results in the sum of derivatives of all classes equal to zero, and the steady state is achieved when $t\to\infty$, although all classes vary with time. This system of equations is not easy to determine the non-trivial (endemic) equilibrium point P^*, and we analyze only the trivial (disease-free) equilibrium point.

The disease-free equilibrium P^0 is given by

$$P^0=\left(s_j^0, q_j^0=0, e_j^0=0, a_j^0=0, q_{1j}^0=0, d_{1j}^0=0, q_{2j}^0=0, d_{2j}^0=0, i^0=0\right),$$

for $j = y, o$, where

$$\begin{cases} s_y^0 = \dfrac{\phi}{\phi + \varphi} \\ s_o^0 = \dfrac{\varphi}{\phi + \varphi}, \end{cases} \tag{20}$$

with $s_y^0 + s_o^0 = 1$.

To assess the stability of P^0, we apply the next generation matrix theory [2]. The next generation matrix is obtained considering the vector of variables $x = (e_y, a_y, d_{1y}, e_o, a_o, d_{1o})$ restricted to the infectious classes of equations (17), (18) and (19). The vectors f and v are constructed considering only equations corresponding to the classes e_y, a_y, d_{1y}, e_o, a_o, and d_{1o}.

In the first approach, we consider the vectors f and v as

$$f^T = \begin{pmatrix} \lambda s_y + e_y \left(\alpha_y d_{2y} + \alpha_o d_{2o}\right) \\ p_y \sigma_y e_y + a_y \left(\alpha_y d_{2y} + \alpha_o d_{2o}\right) \\ \left(1 - p_y\right) \sigma_y e_y + d_{1y} \left(\alpha_y d_{2y} + \alpha_o d_{2o}\right) \\ \lambda \psi s_o + e_o \left(\alpha_y d_{2y} + \alpha_o d_{2o}\right) \\ p_o \sigma_o e_o + a_o \left(\alpha_y d_{2y} + \alpha_o d_{2o}\right) \\ \left(1 - p_o\right) \sigma_o e_o + d_{1o} \left(\alpha_y d_{2y} + \alpha_o d_{2o}\right) \end{pmatrix} \tag{21}$$

and

$$v^T = \begin{pmatrix} \left(\sigma_y + \phi\right) e_y \\ \left(\gamma_y + \eta_y + \chi_y + \phi\right) a_y \\ \left(\gamma_{1y} + \eta_{1y} + \phi\right) d_{1y} \\ \left(\sigma_o + \phi\right) e_o \\ \left(\gamma_o + \eta_o + \chi_o + \phi\right) a_o \\ \left(\gamma_{1o} + \eta_{1o} + \phi\right) d_{1o} \end{pmatrix}, \tag{22}$$

where the superscript T stands for the transposition of a matrix. From these vectors, we obtain the matrices F and V (see [2] for the mathematical procedure), where V is a diagonal matrix. The next generation matrix FV^{-1} evaluated at the trivial equilibrium P^0 is

$$FV^{-1} = \begin{bmatrix} 0 & \frac{\beta_{1y} s_y^0}{\gamma_y + \eta_y + \chi_y + \phi} & \frac{\beta_{2y} s_y^0}{\gamma_{1y} + \eta_{1y} + \phi} & 0 & \frac{\beta_{1o} s_y^0}{\gamma_o + \eta_o + \chi_o + \phi} & \frac{\beta_{2o} s_y^0}{\gamma_{1o} + \eta_{1o} + \phi} \\ \frac{p_y \sigma_y}{\sigma_y + \phi} & 0 & 0 & 0 & 0 & 0 \\ \frac{(1 - p_y) \sigma_y}{\sigma_y + \phi} & 0 & 0 & 0 & 0 & 0 \\ 0 & \frac{\beta_{1y} \psi s_o^0}{\gamma_y + \eta_y + \chi_y + \phi} & \frac{\beta_{2y} \psi s_o^0}{\gamma_{1y} + \eta_{1y} + \phi} & 0 & \frac{\beta_{1o} \psi s_o^0}{\gamma_o + \eta_o + \chi_o + \phi} & \frac{\beta_{2o} \psi s_o^0}{\gamma_{1o} + \eta_{1o} + \phi} \\ 0 & 0 & 0 & \frac{p_o \sigma_o}{\sigma_o + \phi} & 0 & 0 \\ 0 & 0 & 0 & \frac{(1 - p_o) \sigma_o}{\sigma_o + \phi} & 0 & 0 \end{bmatrix},$$

and the characteristic equation corresponding to FV^{-1} is

$$\omega^4 \left(\omega^2 - R_i\right) = 0, \tag{23}$$

where R_i is the reproduction number with intervention given by

$$R_i = R_{iy} s_y^0 + R_{io} s_o^0, \quad \text{where} \quad \left\{ \begin{array}{l} R_{iy} = p_y R_{iy}^1 + (1 - p_y) R_{iy}^2 \\ R_{io} = \left[p_o R_{io}^1 + (1 - p_o) R_{io}^2\right] \psi, \end{array} \right. \tag{24}$$

and R_{iy} and R_{io} are the partial reproduction numbers defined by

$$\left\{ \begin{array}{l} R_{iy}^1 = \dfrac{\sigma_y}{\sigma_y + \phi} \dfrac{\beta_{1y}}{\gamma_y + \eta_y + \chi_y + \phi}, \quad \text{and} \quad R_{iy}^2 = \dfrac{\sigma_y}{\sigma_y + \phi} \dfrac{\beta_{2y}}{\gamma_{1y} + \eta_{1y} + \phi} \\ R_{io}^1 = \dfrac{\sigma_o}{\sigma_o + \phi} \dfrac{\beta_{1o}}{\gamma_o + \eta_o + \chi_o + \phi}, \quad \text{and} \quad R_{io}^2 = \dfrac{\sigma_o}{\sigma_o + \phi} \dfrac{\beta_{2o}}{\gamma_{1o} + \eta_{1o} + \phi}. \end{array} \right. \tag{25}$$

To assess the stability of the equilibrium point P^0, we apply the method proposed in [3] and proved in [4], instead of the spectral radius $(\rho\left(FV^{-1}\right) = \sqrt{R_i})$. Hence, the sum of the coefficients of the characteristic equation is the threshold R_i, and P^0 is locally asymptotically stable (LAS) if $R_i < 1$.

In the second approach, we consider the vectors f and v given by

$$\left\{ \begin{array}{l} f = \left(\lambda s_y, 0, 0, \lambda \psi s_o, 0, 0\right) \\ \text{and} \\ v^T = \left(\begin{array}{c} \left(\sigma_y + \phi\right) e_y - e_y \left(\alpha_y d_{2y} + \alpha_o d_{2o}\right) \\ -p_y \sigma_y e_y + \left(\gamma_y + \eta_y + \chi_y + \phi\right) a_y - a_y \left(\alpha_y d_{2y} + \alpha_o d_{2o}\right) \\ -\left(1 - p_y\right) \sigma_y e_y + \left(\gamma_{1y} + \eta_{1y} + \phi\right) d_{1y} - d_{1y} \left(\alpha_y d_{2y} + \alpha_o d_{2o}\right) \\ \left(\sigma_o + \phi\right) e_o - e_o \left(\alpha_y d_{2y} + \alpha_o d_{2o}\right) \\ -p_o \sigma_o e_o + \left(\gamma_o + \eta_o + \chi_o + \phi\right) a_o - a_o \left(\alpha_y d_{2y} + \alpha_o d_{2o}\right) \\ -\left(1 - p_o\right) \sigma_o e_o + \left(\gamma_{1o} + \eta_{1o} + \phi\right) d_{1o} - d_{1o} \left(\alpha_y d_{2y} + \alpha_o d_{2o}\right) \end{array} \right), \end{array} \right.$$

from which we obtain the matrices F and V, where V is the matrix with the most number of non-zero elements. The next generation matrix FV^{-1} evaluated at the trivial equilibrium P^0

is

$$FV^{-1} = \begin{bmatrix} R_{iy}s_y^0 & \frac{\beta_{1y}s_y^0}{\gamma_y+\eta_y+\chi_y+\phi} & \frac{\beta_{2y}s_y^0}{\gamma_{1y}+\eta_{1y}+\phi} & R_{io}s_y^0 & \frac{\beta_{1o}s_y^0}{\gamma_o+\eta_o+\chi_o+\phi} & \frac{\beta_{2o}s_y^0}{\gamma_{1o}+\eta_{1o}+\phi} \\ 0 & 0 & 0 & 0 & 0 & 0 \\ 0 & 0 & 0 & 0 & 0 & 0 \\ R_{iy}s_o^0 & \frac{\beta_{1y}\psi s_o^0}{\gamma_y+\eta_y+\chi_y+\phi} & \frac{\beta_{2y}\psi s_o^0}{\gamma_{1y}+\eta_{1y}+\phi} & R_{io}s_o^0 & \frac{\beta_{1o}\psi s_o^0}{\gamma_o+\eta_o+\chi_o+\phi} & \frac{\beta_{2o}\psi s_o^0}{\gamma_{1o}+\eta_{1o}+\phi} \\ 0 & 0 & 0 & 0 & 0 & 0 \\ 0 & 0 & 0 & 0 & 0 & 0 \end{bmatrix},$$

and the characteristic equation corresponding to FV^{-1} is

$$\omega^5 (\omega - R_i) = 0.$$

In this case, the spectral radius is $\rho(FV^{-1}) = R_i = R_{iy}s_y^0 + R_{io}s_o^0$ given by equation (24), which is equal to the sum of the coefficients. Hence, P^0 is LAS if $\rho < 1$.

Notice that in the absence of interventions, that is, letting in the equations (24) and (25) $\eta_j = \eta_{1j} = \chi_j = \xi_j = \theta_j = 0$ for $j = y, o$, we have the basic reproduction number R_0 given by

$$R_0 = R_{0y}s_y^0 + R_{0o}s_o^0, \quad \text{where} \quad \begin{cases} R_{0y} = p_y R_{0y}^1 + (1-p_y) R_{0y}^2 \\ R_{0o} = \left[p_o R_{0o}^1 + (1-p_o) R_{0o}^2\right]\psi, \end{cases} \tag{26}$$

where R_{0y} and R_{0o} are given by

$$\begin{cases} R_{0y}^1 = \dfrac{\sigma_y}{\sigma_y+\phi}\dfrac{\beta_{1y}}{\gamma_y+\phi}, & \text{and} \quad R_{0y}^2 = \dfrac{\sigma_y}{\sigma_y+\phi}\dfrac{\beta_{2y}}{\gamma_{1y}+\phi} \\ R_{0o}^1 = \dfrac{\sigma_o}{\sigma_o+\phi}\dfrac{\beta_{1o}}{\gamma_o+\phi}, & \text{and} \quad R_{0o}^2 = \dfrac{\sigma_o}{\sigma_o+\phi}\dfrac{\beta_{2o}}{\gamma_{1o}+\phi}. \end{cases} \tag{27}$$

In the above two constructions of vectors f and v, we obtained the same threshold R_i (R_0 in the absence of interventions). Hence, according to [5], the inverse of the basic reproduction number R_0 given by equation (26) is a function of the fraction of susceptible individuals at the endemic equilibrium s^* through

$$f\left(s^*, s_y^*, s_o^*\right) = \frac{1}{R_0} = \frac{1}{R_{0y}s_y^0 + R_{0o}s_o^0}, \tag{28}$$

where $s^* = s_y^* + s_o^*$ (see [5] and [6]). For this reason, the effective reproduction number R_{ef} [7], which varies with time, can not be defined either by $R_{ef} = R_0(s_y + s_o)$, nor $R_{ef} = R_{0y}s_y + R_{0o}s_o$. The function $f(\varkappa)$ is determined by calculating the coordinates of the non-trivial equilibrium point P^*. For instance, for the dengue transmission model, $f(s_1^*, s_2^*) = s_1^* \times s_2^*$, where s_1^* and s_2^* are the fractions at equilibrium of, respectively, human and mosquito populations [6]. For

the tuberculosis model considering drug-sensitive and resistant strains, there is not $f\left(\varkappa\right)$, but s^* is the solution of a second degree polynomial [5].

From equation (28), let us assume that $f\left(s^*, s_y^*, s_o^*\right) = s_y^* + s_o^*$. Then, we can define the approximated effective reproduction number R_{ef} as

$$R_{ef} = R_0\left(s_y + s_o\right), \tag{29}$$

which depends on time, and at the steady state ($R_{ef} = 1$), we have $s^* = 1/R_0$.

The partial reproduction number $R_{0y}^1 s_y^0$ (or $R_{0y}^2 s_o^0$) is the secondary cases produced by an asymptomatic individual (or pre-diseased individual) in a completely susceptible young subpopulation without control, and the partial reproduction number $R_{0o}^1 s_o^0$ (or $R_{0o}^2 s_o^0$) is the secondary cases produced by an asymptomatic individual (or pre-diseased individual) in a completely susceptible elder subpopulation without control. If all parameters are equal, and $\psi = 1$, then

$$R_0 = \left[pR_0^1 + (1-p)\,R_0^2\right],$$

where $R_0^1 = R_{0y}^1 s_y^0 + R_{0o}^1 s_o^0$ and $R_0^2 = R_{0y}^2 s_y^0 + R_{0o}^2 s_o^0$ are the partial reproduction numbers.

The basic reproduction number R_0 given by equation (26) depends on the parameters related to the natural history of CoViD-19 and the transmission rates. However, the model parameters are not accurate, and it is expected that R_0 is influenced by the inaccuracy of those values. The variation of R_0 with uncertainties in the parameters can be assessed by the sensitivity analysis [8].

3 Model parameters estimation

The results obtained in the foregoing section are applied to describe the new coronavirus infections in São Paulo State, Brazil. A traveler returning from Italy on 21 February 2020 was the first case of CoViD-19, which was confirmed on 26 February. The first death due to CoViD-19 was a 62 years old male with comorbidity who never travelled abroad, hence considered as autochthonous transmission. He manifested the first symptoms on 10 March, was hospitalized on 14 March, and died on 16 March. On 24 March, São Paulo State implemented the isolation of persons in non-essential activities and all students until 6 April. The isolation was extended to 22 April and postponed to 10 May.

Let us determine the initial conditions supplied to the system of equations (3), (4), and (6). São Paulo State has $N(0) = N_0 = 44.6 \times 10^6$ inhabitants with 15.3% of elder subpopulation [9]. The value of parameter φ given in Table 2 in the main text was calculated from the

equation (20), $\varphi = b\phi/(1-b)$, where $b = 0.153$ is the proportion of elder persons, and we obtain $\varphi = 6.7 \times 10^{-6}\ days^{-1}$. Hence, the initial conditions for susceptible persons are $S_y(0) = N_y(0) = N_{0y} = 37.8 \times 10^6$ ($\bar{s}_y^0 = N_{0y}/N_0 = 0.848$) and $S_o(0) = N_o(0) = N_{0o} = 6.8 \times 10^6$ ($\bar{s}_o^0 = N_{0o}/N_0 = 0.152$). For other variables, using $p_y = 0.8$ and $m_y = 0.8$ from Table 2, the ratio asymptomatic:symptomatic is $4:1$, and the ratio mild:severe (non-hospitalized:hospitalized) CoViD-19 is $4:1$. We also use these ratios for elder persons, although $p_o = 0.75$ and $m_o = 0.75$. Hence, if we assume that there is one person in D_{2j} (the first confirmed case), then there are 4 persons in Q_{2j}. The sum (5) is the number of persons in class D_{1j}, implying that there are 20 in class A_j, hence, the sum (25) is the number of persons in class E_j. Finally, at the beginning of the epidemic, there are not isolated, tested, and immunized persons.

Therefore, the trajectories of the new coronavirus propagation are obtained by evaluating the system of equations (3), (4), and (6) numerically using the 4th order Runge-Kutta method, with the initial conditions being given by

$$\begin{cases} S_j(0) = N_{0j}, \quad Q_j(0) = Q_{1j}(0) = 0, \quad E_j(0) = 25, \\ A_j(0) = 20, \quad D_{1j}(0) = 5, \quad Q_{2j}(0) = 4 \quad D_{2j}(0) = 1, \quad I(0) = 0. \end{cases}$$

The initial simulation time $t = 0$ corresponds to the calendar time 26 February 2020, when the first case was confirmed.

To estimate the transmission rates, we assume

$$\beta_y = \beta_{1y} = \beta_{2y} = \beta_{1o} = \beta_{2o}, \qquad \text{and} \qquad \beta_o = \psi\beta_y,$$

that is, all transmission rates in young persons are equal, as well as in elder persons. Hence, the forces of infection are $\lambda_y = (A_y + D_{1y} + A_o + D_{1o})\,\beta_y/N$ and $\lambda_o = \psi\lambda_y$. The reason to include factor ψ is the reduced capacity of a defense mechanism by elder persons (physical barriers, innate and adaptive immune responses, etc.). The force of infection takes into account all virus released by infectious individuals (A_y, D_{1y}, A_o, and D_{1o}), the rate of encounter with susceptible persons, and the capacity to infect them (see [10] [11]). Additionally, the amount inhaled by susceptible persons (especially health care workers) can be determinant in the chance of infection and the prognosis of CoViD-19 [12].

Based on the data collected from São Paulo State, we estimate the transmission (β_y and β_o) and additional mortality (α_y and α_o) rates, and the proportions k_y and k_o of isolated persons. Currently, there is not a sufficient number of kits to detect infection by the new coronavirus. For this reason, tests to confirm infection by this virus are done only in hospitalized persons and those who died manifesting symptoms of CoViD-19. Taking into account hospitalized persons

with CoViD-19 ($\Omega = \Omega_y + \Omega_o$), we fit the transmission rates and the proportion in isolation, and those who died due to CoViD-19 ($\Pi = \Pi_y + \Pi_o$), we fit the additional mortality rates. These parameters can be fitted applying the least square method (see [13]), that is,

$$\min \sum_{i=1}^{n} \left\{ Z_y(t_i) + Z_o(t_i) - \left[Z_y^{ob}(t_i) + Z_o^{ob}(t_i) \right] \right\}^2 , \tag{30}$$

where min stands for the minimum value, n is the number of observations, t_i is the i-th observation time, Z_j stands for Ω_j given by equation (12) and for Π_j given by equation (13), and Z_j^{ob} stands for the observed number of hospitalized persons Ω_j^{ob} and the number of deaths Π_j^{ob}, $j = y, o$.

The least square estimation method is extremely complex, and considering the observed data only in one variable of the dynamic system may not be appropriate (see [14]). For this reason, we evaluate the sum of squared differences by varying the model parameters. This simplified method of parameter evaluation does not provide uncertainties associated with the parameters. The effects of these uncertainties on the epidemic can be assessed by global sensitivity analysis [15], and stochastic simulations can be performed to evaluate the variation in the dynamic variables [16].

3.1 Fitting the transmission rates

The introduction of isolation occurred at $t = 27$, corresponding to the calendar time 24 March, but the effects are expected to appear on 2 April. (The sum of the incubation and pre-diseased infection periods is 9 days, see Table 2 in the main text.) Hence, we will estimate taking into account the confirmed cases of CoViD-19 from 26 February ($t = 0$) to 2 April ($t = 36$), totalizing 37 observations.

To estimate the transmission rates β_y and β_o, we let $\alpha_y = \alpha_o = 0$ and the system of equations (3), (4), and (6), with initial conditions given by equation (7), is evaluated and we calculate

$$\sum_{i=1}^{n} \left\{ \Omega_y(t_i) + \Omega_o(t_i) - \left[\Omega_y^{ob}(t_i) + \Omega_o^{ob}(t_i) \right] \right\}^2$$

by varying β_y and $\beta_o = \psi\beta_y$. The estimated values are $\beta_y = 0.75$ and $\beta_o = 0.88$ (both in $days^{-1}$), where $\Psi = 1.17$, resulting in the basic reproduction number $R_0 = 6.8$ (partials $R_{0y} = 5.6$ and $R_{0o} = 1.2$), according to equation (26). Figure S1(a) shows the estimated curve of Ω and the observed data, plus two curves with lower transmission rates: $\beta_y = 0.55$ and $\beta_o = 0.64$ (both in $days^{-1}$), with $R_0 = 5.0$, and $\beta_y = 0.45$ and $\beta_o = 0.53$ (both in $days^{-1}$), with $R_0 = 4.1$ (partials

$R_{0y} = 3.4$ and $R_{0o} = 0.7$). Figure S1(b) shows the curves of D_2 for young, elder, and total persons for $R_0 = 6.8$ and 4.1, from $t = 30$ until 180.

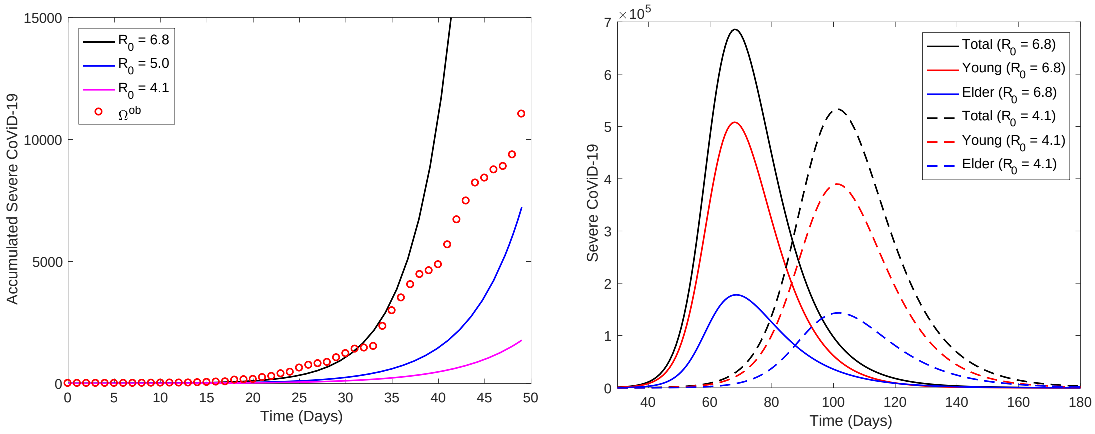

(a) (b)

Figure S1: The estimated curve of severe CoViD-19 cases Ω ($\beta_y = 0.75$ and $\beta_o = 0.88$ (both in $days^{-1}$), with $R_0 = 6.8$) and the observed data, plus two curves with lower transmission rates: $\beta_y = 0.55$ and $\beta_o = 0.64$ (both in $days^{-1}$), with $R_0 = 5.0$; and $\beta_y = 0.45$ and $\beta_o = 0.53$ (both in $days^{-1}$), with $R_0 = 4.1$. Estimation was done using data from $t = 0$ to $t = 39$ (a), and the extended curves of D_2 until $t = 250$ (b). The continuous curve is for $R_0 = 6.8$, and dashed curve for $R_0 = 4.1$.

From Figure S1(a), as R_0 decreases, the estimations become worse. From Figure S1(b), the peaks of the epidemic for young, elder, and total persons are, respectively, 5.08×10^5, 1.78×10^5, and 6.86×10^5 for $R_0 = 6.8$, and the peaks are 3.90×10^5, 1.43×10^5, and 5.33×10^5 for $R_0 = 4.1$. For all persons, the peaks of the epidemic occur at $t = 68$ for $R_0 = 6.8$, and at $t = 101$ for $R_0 = 4.1$.

3.2 Fitting the additional mortality rates

We estimate taking into account confirmed deaths due to CoViD-19 from 16 March ($t = 19$) to 5 April ($t = 39$), totalizing 21 observations.

To estimate the mortality rates α_y and α_o, we fix the previously estimated transmission rates β_y and β_o. The system of equations (3), (4), and (6), with initial conditions given by equation (7), is evaluated and we calculate

$$\sum_{i=1}^{n} \left\{ \Pi_y (t_i) + \Pi_o (t_i) - \left[\Pi_y^{ob} (t_i) + \Pi_o^{ob} (t_i) \right] \right\}^2$$

by varying α_y and α_o. From the fact that fatality among young persons is lower than elder persons, we let $\alpha_y = 0.2\alpha_o$ [17], and estimate only one variable α_o. We estimate the additional mortality rates α_y and α_o for two values of R_0. First, for the transmission rates $\beta_y = 0.75$ and $\beta_o = 0.88$ (both in $days^{-1}$), the estimated mortality rates are $\alpha_y = 0.0052$ and $\alpha_o = 0.026$ (both in $days^{-1}$). For $R_0 = 4.1$, fixing $\beta_y = 0.45$ and $\beta_o = 0.53$ (both in $days^{-1}$), we estimated $\alpha_y = 0.08$ and $\alpha_o = 0.4$ (both in $days^{-1}$). This is called the first estimation method.

The first estimation method used only one information: the risk of death is higher in elders than young persons (we used $\alpha_y = 0.2\alpha_o$). However, the fatality among hospitalized elder persons is around 10% [17]. Combining both findings, we assume that the deaths for young and elder persons are, respectively, 2% and 10% of accumulated cases when Ω_y and Ω_o approach plateaus (see Figures S3(a) and S4(a) below). This is called the second estimation method, which takes into account this second information besides the one used in the first estimation method. In this procedure, the estimated rates are $\alpha_y = 0.0018$ and $\alpha_o = 0.009$ (both in $days^{-1}$) for both R_0.

Figure S2 shows the estimated curves of $\Pi = \Pi_y + \Pi_o$ provided by both methods of estimation and the observed data for $R_0 = 6.8$ (a) and 4.1 (b). The second method of estimation fits badly the interval of estimation from $t = 19$ to 39.

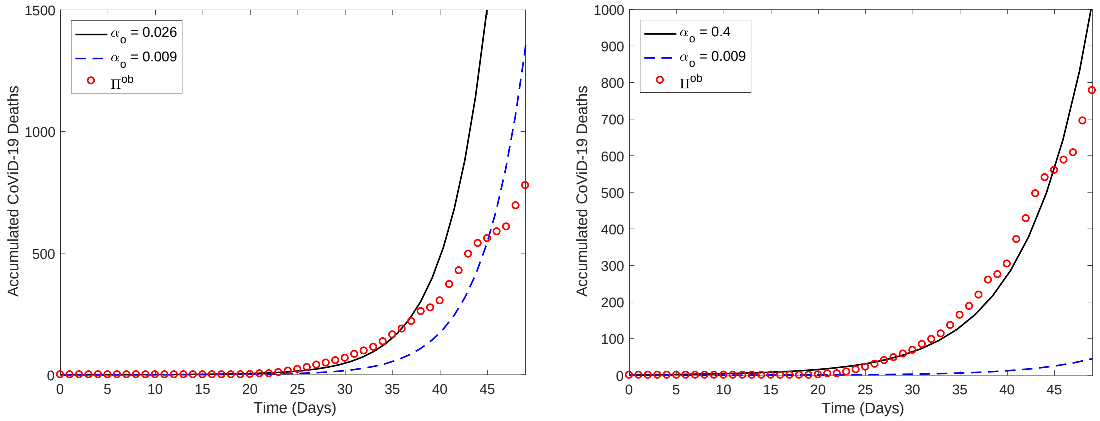

(a) (b)

Figure S2: The estimated curves of Π provided by both methods of estimation and the observed data from $t = 0$ until 39. The first estimation method provided for $R_0 = 6.8$, $\alpha_y = 0.0052$ and $\alpha_o = 0.026$ (a), and for $R_0 = 4.1$, $\alpha_y = 0.08$ and $\alpha_o = 0.4$ (b). Both figures show also the second method of estimation, providing the same $\alpha_y = 0.0009$ and $\alpha_o = 0.009$. The rates α_y and α_o are in $days^{-1}$.

For $R_0 = 6.8$, Figure S3(a) shows the estimated curves of accumulated number of severe CoViD-19 (Ω_y, Ω_o, and $\Omega = \Omega_y + \Omega_o$), from equation (12). At $t = 120$, Ω approached an

asymptote (or plateau), which can be understood as the time when the first wave of the epidemic ends. The curves Ω_y, Ω_o, and Ω reach values, respectively, 1.51×10^6, 0.43×10^6, and 1.94×10^6. Figure S3(b) shows the estimated curves of the accumulated number of CoViD-19 deaths (Π_y, Π_o, and $\Pi = \Pi_y + \Pi_o$), from equation (13). The values of Π_y, Π_o, and Π are, for the first method of estimation, respectively, 0.747×10^5 (4.9%), 1.14×10^5 (26.7%) and 1.88×10^5 (9.7%), and for the second method of estimation, respectively, 2.67×10^4 (1.77%), 4.77×10^4 (11.2%) and 7.44×10^4 (3.8%). The percentage between parentheses is the ratio Π/Ω.

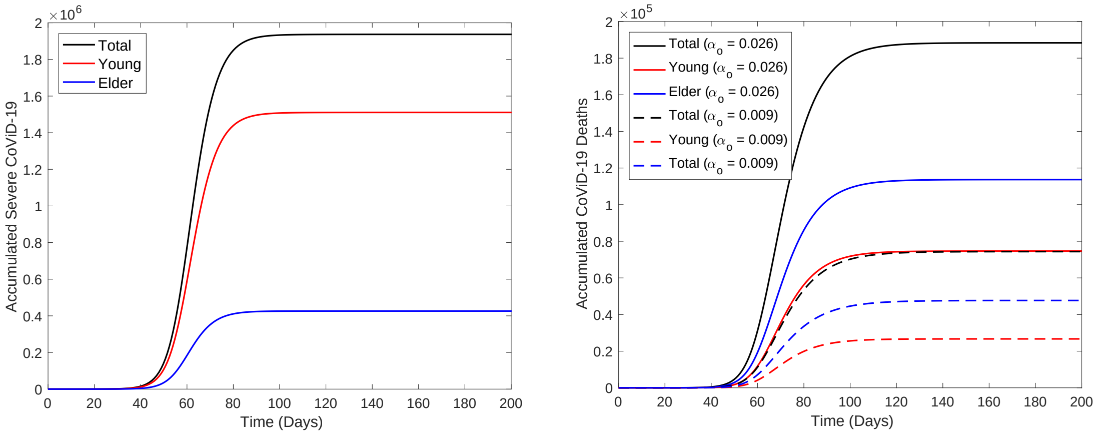

(a) (b)

Figure S3: For $R_0 = 6.8$, the estimated curves of accumulated number of severe CoViD-19 (Ω_y, Ω_o and $\Omega = \Omega_y + \Omega_o$) (a), and accumulated number of CoViD-19 deaths (Π_y, Π_o and $\Pi = \Pi_y + \Pi_o$) (b). The continuous curve for $\alpha_o = 0.026$ $days^{-1}$, and dashed curve for $\alpha_o = 0.009$ $days^{-1}$.

From Figure S3, the first estimation method for the additional mortality rates provided around 2.5-time more deaths than the second method. Especially, the first method estimated 26.7% against 11.2% of deaths in the elder subpopulation, while for the young subpopulation, 5% against 2% in comparison with the second method. Hence, the second estimation method is more credible.

For $R_0 = 4.1$, Figure S4(a) shows the estimated curves of the accumulated number of severe CoViD-19 (Ω_y, Ω_o, and $\Omega = \Omega_y + \Omega_o$), from equation (12). At $t = 200$, the curves Ω_y, Ω_o, and Ω reach values, respectively, 1.48×10^6, 0.42×10^6, and 1.91×10^6. Figure S4(b) shows the estimated curves of the accumulated number of CoViD-19 deaths (Π_y, Π_o, and $\Pi = \Pi_y + \Pi_o$), from equation (13). The values of Π_y, Π_o, and Π are, for the first method of estimation, respectively, 6.60×10^5 (44.4%), 3.58×10^5 (84.9%) and 1.02×10^6 (53.4%), and for the second method of estimation, respectively, 2.62×10^4 (1.8%), 4.72×10^4 (11.2%) and 7.34×10^4 (3.9%). The percentage between parentheses is the ratio Π/Ω.

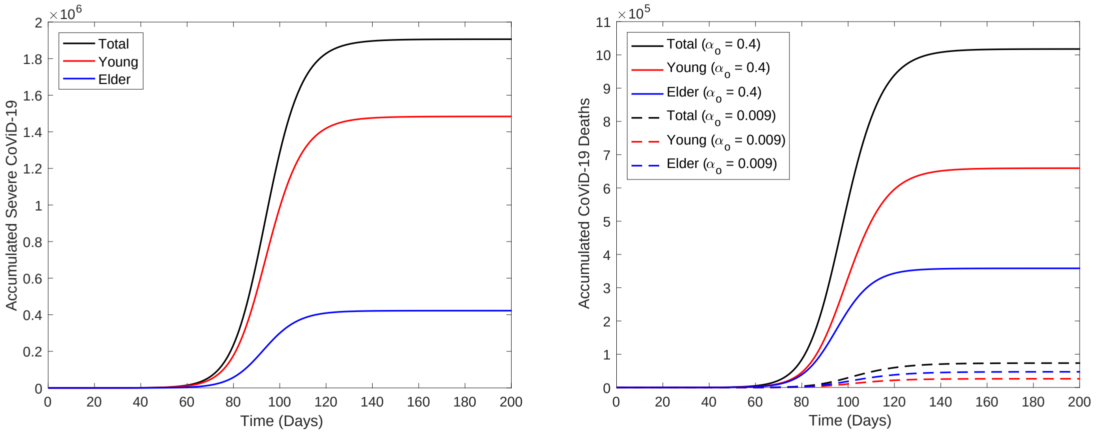

(a) (b)

Figure S4: For $R_0 = 4.1$, the estimated curves of accumulated number of severe CoViD-19 (Ω_y, Ω_o and $\Omega = \Omega_y + \Omega_o$) (a), and accumulated number of CoViD-19 deaths (Π_y, Π_o and $\Pi = \Pi_y + \Pi_o$) (b). The continuous curve for $\alpha_o = 0.4$ $days^{-1}$, and dashed curve for $\alpha_o = 0.009$ $days^{-1}$.

From Figure S4, the first estimation method for the additional mortality rates provided much higher values than the second method, for instance, 25-time more deaths in the young subpopulation. Especially, the first method estimated 85% against 11.2% of deaths in the elder subpopulation in comparison with the second method. Notice that, from Figures S3 and S4, at the end of the first wave of the epidemic, we have quite the same number of accumulated CoViD-19 cases for $R_0 = 6.8$ and 4.1 (difference at most 1.8%) despite big differences in the accumulated deaths.

3.3 Estimation of the proportion in isolation

Isolation was introduced at $t = 27$ (24 March), and we estimate k_y and k_o taking into account the confirmed cases of CoViD-19 from $t = 27$ to 55 (21 April), totalizing 29 observations.

To estimate the proportion in isolation, we vary k letting $k = k_y = k_o$. The system of equations (3), (4), and (6), with boundary conditions given by equations (8) and (9), is evaluated and we calculate

$$\sum_{i=1}^{n} \left\{ \Omega_y(t_i) + \Omega_o(t_i) - \left[\Omega_{2y}^{b}(t_i) + \Omega_o^{ob}(t_i) \right] \right\}^2 ,$$

where $t_1 = 27$ and $t_{29} = 55$. We varied k, $k = 0$, 0.4, 0.6, 0.7, and 0.8, and we choose as the estimated value $k = 0.5$, which is close to the observed proportions of isolation in São Paulo

State [18].

Figure S5(a) shows the estimated curve of $\Omega = \Omega_y + \Omega_o$ and the observed data, plus the curves for $k = 0$, 0.4, 0.6, 0.7, and 0.8. Figure S5(b) shows the curves of D_2 for 6 different values of k, extended from $t = 0$ until 250. For $k = 0$, 0.4, 0.5, 0.6, 0.7, and 0.8, the peaks of the epidemic are, respectively, 6.89×10^5, 3.12×10^5, 2.25×10^5, 1.42×10^5, 0.68×10^5, and 0.14×10^5, which decrease as k increases, and displace to the right to, respectively, $t = 68$, 82, 88, 99, 118, and 165.

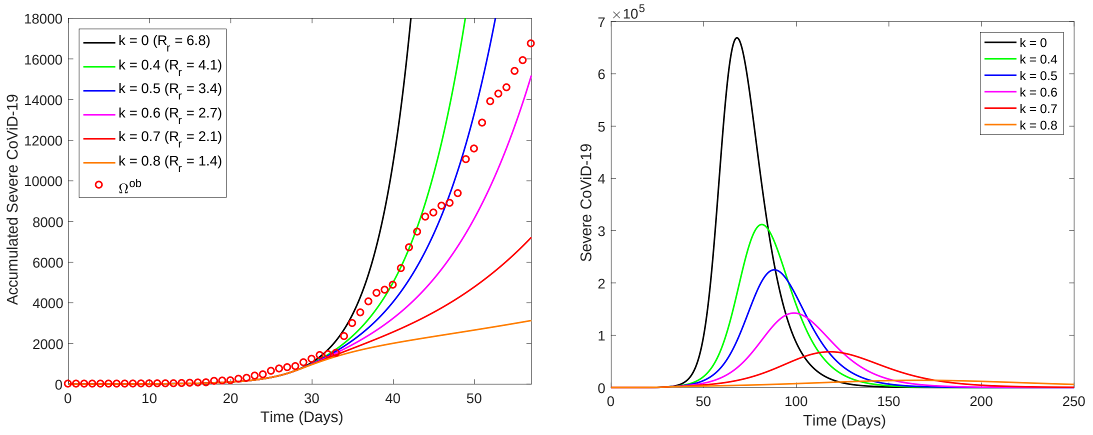

(a) (b)

Figure S5: The estimated curve of Ω for $k = 0.5$, and the observed data, plus five curves of k, $k = 0$, 0.4, 0.6, 0.7, and 0.8 (a), and the extended curves of D_2 from $t = 0$ until 250 (b).

Figure S6 shows the curves of Ω_y, Ω_o, and $\Omega = \Omega_y + \Omega_o$ (a) and Π_y, Π_o, and $\Pi = \Pi_y + \Pi_o$ (b) without ($k = 0$, continuous curves) and with ($k = 0.5$, dashed curves) isolation. For $k = 0.5$, at $t = 250$, the curves Ω_y, Ω_o, and Ω attain values, respectively, 7.30×10^5, 2.09×10^5, and 9.38×10^5, and Π_y, Π_o, and Π attain, respectively, 1.29×10^4 (1.77%), 2.33×10^4 (11.17%), and 3.62×10^4 (3.86%). The percentage between parentheses is the ratio Π/Ω.

Figure S7 shows the curves of S_y, S_o, and $S = S_y + S_o$ (a) and I_y, I_o, and $I = I_y + I_o$ (b) without ($k = 0$, continuous curves) and with ($k = 0.5$, dashed curves) isolation. For $k = 0.5$, at $t = 250$, the numbers of susceptible persons S_y, S_o, and $S = S_y + S_o$ are, respectively, 9.95×10^5 (2.63%), 7.94×10^4 (1.17%), and 1.08×10^6 (2.41%). The percentage between parentheses is the ratio $S(250)/S(0)$. The numbers of immune persons I_y, I_o, and I increase from zero to, respectively, 1.81×10^7 (47.9%), 0.33×10^7 (48.4%), and 2.14×10^7 (48%), for $k = 0.5$. The percentage between parentheses is the ratio $I/S(0)$, and $S(0)$, where $S_y(0) = 37.8 \times 10^6$ and $S_o(0) = 6.8 \times 10^6$.

From the foregoing section, we transport the values for $k = 0$ at the end of the first wave

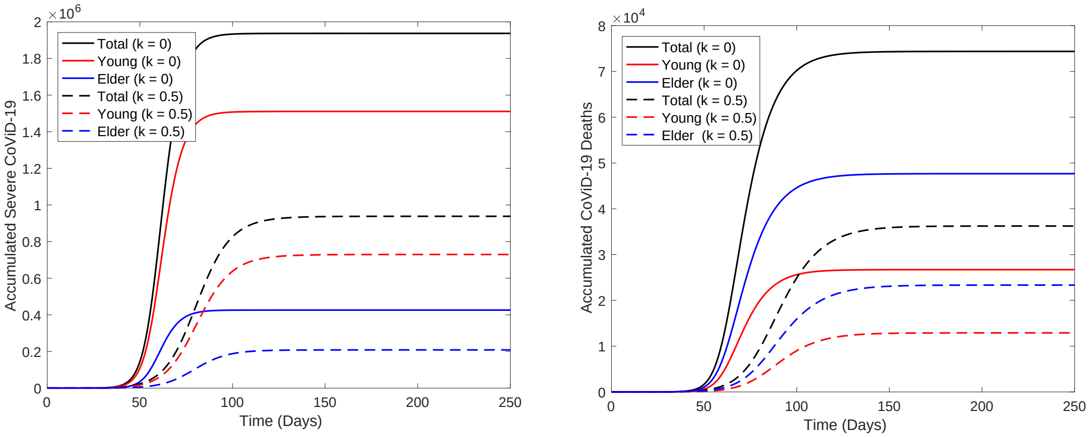

(a) (b)

Figure S6: The curves of Ω_y, Ω_o, and $\Omega = \Omega_y + \Omega_o$ (a), and Π_y, Π_o, and $\Pi = \Pi_y + \Pi_o$ (b) without ($k = 0$, continuous curves) and with (proportion $k = 0.5$, dashed curves) isolation.

of the epidemic. For Ω_y, Ω_o, and Ω, we have 1.51×10^6, 0.43×10^6, and 1.94×10^6, and for Π_y, Π_o, and Π, the values are 2.67×10^4 (1.77%), 4.77×10^4 (11.19%), and 7.44×10^4 (3.84%). The percentage between parentheses is the ratio Π/Ω. For S_y, S_o, and S, we have 2.35×10^5 (0.62%), 0.30×10^4 (0.043%), and 2.38×10^5 (0.53%), and the percentage between parentheses is the ratio $S(200)/S(0)$. For I_y, I_o, and I, we have 3.755×10^7 (99.4%), 0.67×10^7 (98.8%), and 4.43×10^7 (99.3%), and the percentage between parentheses is the ratio $I/S(0)$. At $t = 0$, for S_y, S_o, and S, we have 3.78×10^7, 0.68×10^7, and 4.46×10^7.

For $k = 0.5$, at the end of the first wave of the epidemic, in comparison with $k = 0$, the severe CoViD-19 cases Ω_y, Ω_o, and Ω decreased by 51%, 49%, and 48%, while Π_y, Π_o, and Π decreased by 48%, 49%, and 49%. The susceptible persons S_y, S_o, and S increased by 424%, 2701%, and 453%, while I_y, I_o, and I decreased by 48%, 49%, and 48%. Notice that the numbers of severe CoViD-19, deaths due to CoViD-19, and immune persons decreased by 50%, while susceptible young persons were increased around 4-time (27-time for elder persons). These epidemiological scenarios for $k = 0.5$ show that the elder subpopulation will be at a higher risk of infection in the second wave of the epidemic.

To assess the effects of isolation on the epidemic, we consider $k = 0.6$, 0.7, and 0.8. As k increases (assuming 0.6, 0.7, and 0.8), the peaks of D_2 decrease to 142.3, 68, and 13.7 (all in thousand), occurring at 99 (4 June), 118 (23 June) and 165 (9 August), which are delayed in 31, 50, and 97 days in comparison with $k = 0$. Table S1 shows the values of Ω, Π, S, and I at $t = 250$, for $k = 0.6$, 0.7, and 0.8. Percentages are calculated with respect to $k = 0$. As k increases, the numbers of severe CoViD-19 and deaths decrease, which help in the hospital

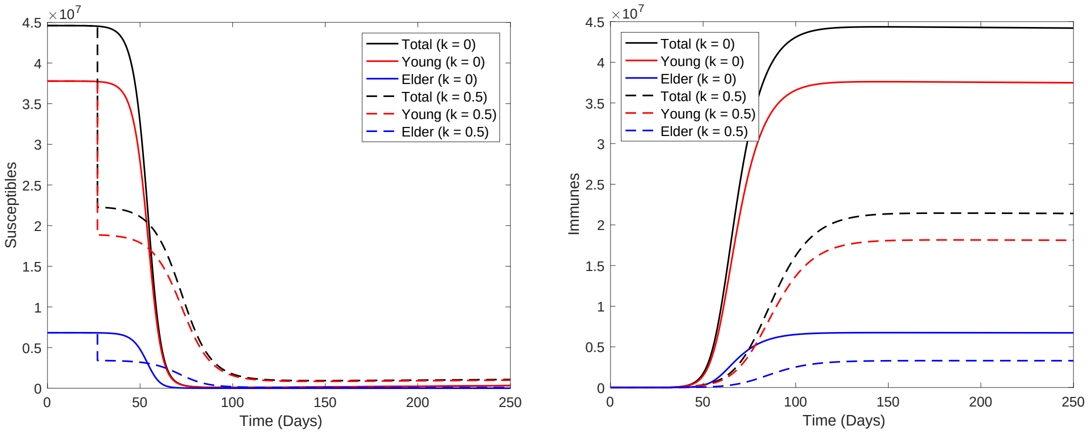

(a) (b)

Figure S7: The curves of S_y, S_o, and $S = S_y + S_o$ (a), and I_y, I_o, and $I = I_y + I_o$ (b) without ($k = 0$, continuous curves) and with (proportion $k = 0.5$, dashed curves) isolation.

management and health care system. However, the number of susceptible persons increases and fewer persons are immune at the end of the first wave of the epidemic as k increases, which indicates more risk of the second wave of the epidemic, especially in elder subpopulation.

Table S1: The values of Ω, Π, S, and I at $t = 250$, for $k = 0.6$, 0.7, and 0.8. y, o, and Σ stand for, respectively, young, elder, and total persons.

	$k = 0.6$			$k = 0.7$			$k = 0.8$		
	y	o	Σ	y	o	Σ	y	o	Σ
Ω (10^5)	5.58	1.62	7.20	3.72	1.10	4.82	1.43	0.44	1.87
Π	9866	18060	27926	6568	12270	18838	2451	4752	7203
S (10^6)	1.51	0.15	1.66	2.39	0.30	2.69	4.24	0.65	4.90
I (10^6)	13.86	2.55	16.41	9.24	1.74	10.97	3.50	0.68	4.18
Ω (%)	36.94	37.89	37.15	24.61	25.79	24.87	9.48	10.37	9.67
Π (%)	36.95	37.89	37.55	24.60	25.74	25.33	9.18	9.97	9.69
S (%)	477	5055	520	755	9845	841	1342	21606	1534
I (%)	36.98	37.82	37.11	24.65	25.73	24.81	9.32	10.13	9.45

Table S1 summarized the epidemic in the circulating population when a proportion k of the population is isolated. In comparison with the epidemic without isolation, at the end of the first wave of the epidemic, the numbers of severe CoViD-19 cases Ω, deaths Π, and immune persons I were decreased by around $100k\%$, while susceptible persons were increased. For $k = 0.5$, 0.6, 0.7, and 0.8, the susceptible young persons have increased by around, respectively, 4, 5, 7, and 13-time, while for the susceptible elder persons, 27, 50, 100, and 216-time. The effect of

isolation to control the epidemic is portrayed by the increased number of susceptible persons at the end of the epidemic. Clearly, the elder subpopulation is highly benefitted from the isolation, much more than the young subpopulation. This finding shows that the interaction between elder and young subpopulations results in much more cases of CoViD-19 in the elder subpopulation, and the isolation protects the more vulnerable elder persons.

We summarize the estimation of the model parameters and the current epidemiological scenario under isolation.

At $t = 0$, the numbers of susceptible persons, S_y, S_o, and $S = S_y + S_o$ are, respectively, 3.78×10^7, 0.68×10^7, and 4.46×10^7, which diminish due to infection. At $t = 200$, for $R_0 = 6.8$, the numbers of susceptible young, elder, and total persons are, respectively, 2.35×10^5 (0.62%), 0.29×10^4 (0.04%), and 2.38×10^5 (0.53%). For $R_0 = 4.1$, the numbers of susceptible persons are 8.82×10^5 (2.34%), 6.96×10^4 (1.02%), and 9.52×10^5 (2.13%). The percentage between parentheses is the ratio $S(200)/S(0)$. The numbers of susceptible young and total persons are 4-time higher when R_0 decreases from 6.8 to 4.1, while for elder persons, it is 24-time.

For $R_0 = 6.8$, at $t = 200$, the numbers of immune persons (I_y, I_o, and I) increase from zero to, respectively, 3.76×10^7 (99.4%), 0.67×10^7 (98.8%), and 4.43×10^7 (99.3%), for young, elder and total persons. For $R_0 = 4.1$, the numbers of immune persons are 3.69×10^7 (97.7%), 0.67×10^7 (97.9%), and 4.36×10^7 (97.7%), for young, elder and total persons, respectively. The percentage between parentheses is the ratio $I/S(0)$.

At the end of the first wave of the epidemic, the values of Ω, Π, S, and I are practically the same for $R_0 = 6.8$ and 4.1. However, the peak of the epidemic for $R_0 = 6.8$ is higher than $R_0 = 4.1$, and the curve of the epidemic for $R_0 = 4.1$ is more spread over time than the curve for $R_0 = 6.8$.

The adoption of isolation in São Paulo State maintained 18.9×10^6 young and 3.4×10^6 elder persons in the susceptible class. These susceptible persons will be joined with those in the circulating population, showing that strategies of release must be planned carefully. In the main text, the estimated model parameters are used to understand the current epidemiological scenario of the new coronavirus in São Paulo State, and to evaluate the release of isolated persons.

To evaluate how the model parameters affect the epidemic, we varied the estimated values. Two decreased values in the transmission rates, resulting in $R_0 = 5$ and $R_0 = 4.1$, underestimated the curves of the epidemic (see Figure S1(a)). The estimated additional mortality rates fitting very well the curve of deaths at the beginning of the epidemic provided an unrealistic number of deaths at the end of the first wave of the epidemic. We chose decreased mortality rates to obtain a more realistic estimation of deaths at the end of the epidemic (see Figures

S2-S4). We varied by 10% the estimated proportion in isolation, and we obtained an underestimated curve of the epidemic for $k = 0.6$, but good fitting for $k = 0.4$. However, we choose $k = 0.5$ based on the observed proportions in isolation in São Paulo State (see Figure S5).

References

[1] **Anderson RM and May RM**. *Infectious Diseases of Human. Dynamics and Control*, Oxford, New York, Tokyo: Oxford University Press, 1991, pp. 757.

[2] **Diekmann O, Heesterbeek JAP and Roberts MG**. The construction of next-generation matrices for compartmental epidemic models. *Journal of The Royal Society Interface* 2010; **7**: 873-885.

[3] **Yang HM**. The basic reproduction number obtained from Jacobian and next generation matrices – A case study of dengue transmission modelling. *BioSystems* 2014; **126**: 52-75.

[4] **Yang HM and Greenhalgh D**. Proof of conjecture in: The basic reproduction number obtained from Jacobian and next generation matrices – A case study of dengue transmission modelling. *Applied Mathematics and Computation* 2015; **265**: 103-107.

[5] **Yang HM**. Are the beginning and ending phases of epidemics provided by next generation matrices? – Revisiting drug sensitive and resistant tuberculosis model. *ArXiv.org* (*www.arxiv.org*) 2020; pp 18 (https://arxiv.org/abs/2006.06857v1).

[6] **Yang HM**. The transovarial transmission un the dynamics of dengue infection: Epidemiological implications and thresholds. *Mathematical Biosciences* 2017; **286**: 1-15.

[7] **Yang HM, et al**. Fitting the incidence data from the City of Campinas, Brazil, based on dengue transmission modellings considering time-dependent entomological parameters. *PlosOne* 2016; **March 24**: 1-41.

[8] **Yang HM**. A mathematical model for malaria transmission relating global warming and local socioeconomic conditions. *Revista de Saúde Pública* 2001; **35**: 224-231.

[9] **Fundação Sistema Estadual (SEADE) database** (https://www.seade.gov.br). Accessed 9 April 2020.

[10] **Yang HM**. Directly transmitted infections modeling considering age-structured contact rate – Epidemiological analysis. *Mathematical and Computer Modelling* 1999; **29**: 11-30.

[11] **Yang HM**. Directly transmitted infections modeling considering age-structured contact rate. *Mathematical and Computer Modelling* 1999; **29**: 39-48.

[12] **Gomez MC and Yang HM**. A simple mathematical model to describe antibody-dependent enhancement in heterologous secondary infection in dengue. *Mathematical Medicine and Biology: A Journal of the IMA* 2019; **36**: 411–438.

[13] **Raimundo SM, et al**. The attracting basins and the assessment of the transmission coefficients for HIV and *M. tuberculosis* infections among women inmates. *Journal of Biological Systems* 2002; **10**: 61-83.

[14] **Bates DM and Watts DG**. *Nonlinear Regression Analysis and Its Applications*, New York: John Willey and Sons, 1988, pp. 365.

[15] **Marino S, et al**. A methodology for performing global uncertainty and sensitivity analysis in systems biology. *Journal of theoretical biology* 2008; **254**: 178-196.

[16] **Freitas LFS.** Vacinação de doenças infecciosas de transmissão direta: quantificando condições de controle considerando portadores (PhD Thesis). Campinas, SP, Bazil: The State University at Campinas, 2018, 171 pp. Available from: http://www.repositorio.unicamp.br/handle/REPOSIP/331644.

[17] **Boletim Epidemiológico (BE) 08 database** (https://www.saude.gov.br/images/pdf/2020/April/09/be-covid-08-final-2.pdf). Accessed 9 April 2020.

[18] **Adesão ao isolamento social em SP (AIS) database** (https://www.saopaulo.sp.gov.br/coronavirus/isolamento). Accessed 9 April 2020.
